# Infant word recognition: Insights from TRACE simulations^☆^

**DOI:** 10.1016/j.jml.2013.09.009

**Published:** 2014-02

**Authors:** Julien Mayor, Kim Plunkett

**Affiliations:** a FPSE, University of Geneva, Switzerland; b Department of Experimental Psychology, University of Oxford, United Kingdom

**Keywords:** Phonological specificity, Vowels, Consonants, Lexical representation, Lexical competition, TRACE model

## Abstract

The TRACE model of speech perception (McClelland & Elman, 1986) is used to simulate results from the infant word recognition literature, to provide a unified, theoretical framework for interpreting these findings. In a first set of simulations, we demonstrate how TRACE can reconcile apparently conflicting findings suggesting, on the one hand, that consonants play a pre-eminent role in lexical acquisition (Nespor, Peña & Mehler, 2003; Nazzi, 2005), and on the other, that there is a symmetry in infant sensitivity to vowel and consonant mispronunciations of familiar words (Mani & Plunkett, 2007). In a second series of simulations, we use TRACE to simulate infants’ graded sensitivity to mispronunciations of familiar words as reported by White and Morgan (2008). An unexpected outcome is that TRACE fails to demonstrate graded sensitivity for White and Morgan’s stimuli unless the inhibitory parameters in TRACE are substantially reduced. We explore the ramifications of this finding for theories of lexical development. Finally, TRACE mimics the impact of phonological neighbourhoods on early word learning reported by Swingley and Aslin (2007). TRACE offers an alternative explanation of these findings in terms of mispronunciations of lexical items rather than imputing word learning to infants. Together these simulations provide an evaluation of Developmental (Jusczyk, 1993) and Familiarity (Metsala, 1999) accounts of word recognition by infants and young children. The findings point to a role for both theoretical approaches whereby vocabulary structure and content constrain infant word recognition in an experience-dependent fashion, and highlight the continuity in the processes and representations involved in lexical development during the second year of life.

## Highlights

• We reconcile conflicting theories about vowels and consonants in early perception.
• Mispronunciation sensitivity is modulated by the size and structure of the lexicon.
• Asymmetries in mispronunciation can be explained with a fully specified phonology.
• Inhibition at a phoneme level and/or at a lexical level is likely reduced in infancy.
• Claim that words from dense neighbourhoods are harder to learn needs a stronger test.

## Introduction

Research on infant spoken word recognition has made dramatic advances over the past two decades. Spurred on by the refinement of experimental techniques such as the familiarisation head turn preference procedure (Jusczyk & Aslin, 1995), the switch task (Stager & Werker, 1997) and the mispronunciation task (Swingley & Aslin, 2000), our understanding of *what* infants and young children know about the sounds of words, both familiar and newly learnt, has expanded incrementally. These studies suggest that rather detailed information about the sounds of familiar words is encoded by 18-month-olds, though less information is encoded for newly learnt words (see Werker & Curtin, 2005, for a review).

Our appreciation of the nature of the representations and processes underlying early phono-lexical knowledge and *how* these develop is less advanced. Two approaches can be broadly distinguished. Developmental accounts highlight the importance of vocabulary structure in sculpting young children’s knowledge about the sounds of words. For example, Jusczyk (1993) argued that the most efficient representation for a word would have just enough detail to achieve successful recognition. On this view, children’s knowledge about the sounds of words should be greatest for words in dense neighbourhoods, so that segments of sound needed to discriminate otherwise similar sequences would be encoded in more detail than non-overlapping sequences. An alternative view is that children’s knowledge about the sounds of words depends on their *familiarity* with particular words rather than the number or density of words in their vocabularies. For example, Metsala (1999) argued that words entering the early lexicon achieve more adult-like representations before words learned at a later age, either owing to familiarity (i.e., frequency of exposure) or to age-of-acquisition effects. The familiarity hypothesis predicts that very young children and older children will have difficulty making fine phonological distinctions for recently learned words, but will be able to make finer distinctions for more familiar words learned at a very young age. Both developmental and familiarity accounts may play a role in constraining how children learn about the sounds of words. For example, Werker and Curtin’s (2005) PRIMIR model proposes a qualitative change between infant and adult representations of words, involving a shift from early phonetic categories to later higher level phonemic categories. Overlap between the sounds of words and item salience are attributed a primary role in driving this change.

### Using TRACE in a visual world

Although these approaches offer important insights as to how infants and young children develop knowledge about the sounds of words, they do not provide a precise computational account of the representations and processes involved. In this paper, we describe our attempt to apply the TRACE model of word recognition (McClelland & Elman, 1986) to simulate aspects of spoken word recognition during infancy and early childhood. TRACE was originally proposed as a model of adult spoken word recognition. In TRACE, spoken word recognition is modelled as an incremental process involving the elimination of competing candidates that are represented in the individual’s mental lexicon. Detailed empirical studies have documented the role of cohort competitors (Marlsen-Wilson & Welsh, 1978) and phonological neighbours (Goldinger et al., 1989, Miller and Eimas, 1995) in this competition. Allopenna, Magnuson, and Tanenhaus (1998) have argued that the TRACE model of speech perception provides a satisfactory accommodation of the role of cohorts and phonological neighbours in the resolution of the competitive process.

Allopenna et al. (1998) used TRACE to simulate findings from an experiment designed to measure spoken word recognition in a visual world task: Adults heard spoken instructions to move one of four objects that were on a screen, while they were simultaneously monitored by an eye-tracker. Along with the target, three competitors were displayed on screen; a cohort competitor (object label starting with the same onset and vowel), a rhyme competitor and an unrelated competitor. Allopenna et al. (1998) found that the likelihood of fixating each item over time depended on their phonological similarity to the target. Just after word onset, participants fixated the target and cohort competitor — items that matched the word onset. They also fixated the rhyme significantly more than the unrelated item, although the effect was delayed and smaller. Using the TRACE model, supplemented with Luce’s choice rule (Luce, 1959) to simulate picture preference, Allopenna et al. (1998) accurately reproduced the typical pattern of eye-gaze of the participants. In particular, TRACE exhibited enhanced activation for cohort and rhyme competitors, resulting in enhanced levels of “eye fixations” in this simulation of the visual world task.

Many other studies have explored the set of parameters in TRACE required to match human experimental data. For example, McClelland and Elman (1986) addressed several cases of mispronunciations and lexical effects. Frauenfelder, Scholten, and Content (2001) discussed the role of bottom-up inhibition in mispronunciation studies and Dahan, Magnuson, Tanenhaus, and Hogan (2001b) evaluated the role of lexical inhibition in subcategorical mismatches. More recently, TRACE has been used to model adults’ gradient sensitivity to within-category voice onset time manipulations in a visual world task (McMurray, Tanenhaus, & Aslin, 2009) and individual differences in online spoken word recognition, including individuals at risk for specific language impairment (McMurray, Samelson, Lee, & Tomblin, 2010). In these applications, exploration of TRACE’s parameter space identified factors (phoneme inhibition, lexical inhibition, lexicon size, and many more) that might account for observed variations in human performance.

TRACE also possesses properties that make it relevant to the investigation of word recognition during earlier stages of development. For example, it is possible to investigate the impact of lexical dynamics in TRACE by manipulating the size and composition of its lexicon. Manipulation of the size and composition of TRACE’s lexicon can be conducted in a fashion that mimics the infant’s developing vocabulary, thereby permitting an evaluation of the impact of vocabulary growth on infant word recognition. It is also possible to investigate the impact of a word’s token frequency in TRACE by manipulating the strength of connections associated with a given word. Each of these manipulations permit a precise evaluation of developmental (increasing vocabulary size) and familiarity (word token frequency) effects on infant spoken word recognition. As a starting point for the current investigation, adult-like connectivity and dynamics are assumed when simulating infant speech perception. The applicability of these assumptions are then refined as we attempt to capture a wider variety of empirical findings from the infant word recognition literature within the framework of TRACE.

TRACE assumes that a word’s phonological form is based on a featural representation that is fully-specified. This assumption fits well with studies using the mispronunciation task reporting that infants can detect single feature deviations from the correct pronunciation of familiar words (Swingley and Aslin, 2000, Swingley and Aslin, 2002). The assumption sits less well with theories that assume phonologically underspecified lexicons (Lahiri & Reetz, 1999). Our research strategy is to take a computational model of spoken word recognition that has been applied successfully to a wide range of experimental findings in the adult literature and evaluate its applicability to infant word recognition. To the degree that TRACE’s architecture (levels of representation, patterns of connectivity, etc.) and phonological representations are able to successfully simulate aspects of language development through manipulations of vocabulary size and relative frequency, *continuity* in the computational processes and phonological representations underlying word recognition from infancy to adulthood can be inferred.

We will show that TRACE is able to simulate a range of experimental findings from the infant speech recognition literature, with relatively few changes to its computational architecture. The implication of this computational result is that TRACE provides a theoretical framework for accommodating both mature state and developmental aspects of spoken word recognition. This is *not* to imply that TRACE is an *adequate* theoretical framework for spoken word recognition. We know of no computational model in psycholinguistics or developmental psycholinguistics that can claim such a status. However, our results provide a baseline against which other future computational models of the development of spoken word recognition during infancy can be judged.

### Brief overview of simulations

We tailor TRACE to infant speech perception and word recognition by manipulating the size and structure of its lexicon in accordance with published norms of infant vocabulary development. Our initial working hypothesis is that changes in the size and structure of infant vocabulary are sufficient to capture developmental trends and other experimental findings reported in the infant word recognition literature. The construction of the new lexicons requires the introduction of a larger phonemic inventory in TRACE to accommodate the range of words used by infants at different ages. To ensure that this change in phonemic inventory does not dramatically distort the phonological space inhabited by TRACE, nor undermine TRACE’s capacity to capture important findings from the adult speech perception and word recognition literature, we carried out a set of baseline analyses and simulations, including some of the original mispronunciation simulations, with these new lexicons. The results of TRACE’s performance under these conditions are reported in Appendix A. Although we have not attempted to replicate *all* of the original simulations conducted by McClelland and Elman (1986), these results indicate that the central performance characteristics remain intact with our new lexicons. All simulations use the jTRACE implementation (Strauss, Harris, & Magnuson, 2007) of the TRACE model.

#### Vowel and consonant mispronunciations

We begin by using TRACE to simulate infants’ sensitivity to vowel and consonant mispronunciations of familiar words, a topic under considerable scrutiny in the past decade. Potentially conflicting findings suggest that consonants play a pre-eminent role in lexical acquisition (Nazzi, 2005, Nespor et al., 2003), while other findings suggest that there is a symmetry in infant sensitivity to vowel and consonant mispronunciations of familiar words (Mani and Plunkett, 2007, Swingley and Aslin, 2000, Swingley and Aslin, 2002). TRACE makes no fundamental distinction in its representation of vowels and consonants. Both types of phonemic segments are uniquely and fully specified across the same set of 7 continuously varying features in TRACE (see Table 1). However, TRACE is sensitive to the identity and position of phonemic segments in a word as well as to its set of cohort and neighbourhood competitors. Hence, although we would expect TRACE to demonstrate sensitivity to both vowel and consonant mispronunciations, the impact of these other factors on different types of mispronunciation sensitivities is far from obvious, particularly when the composition of the lexicon changes over development. Simulation offers an invaluable tool to predict the impact of these changes. We demonstrate how TRACE can reconcile these disparate findings.

**Table 1:** Phoneme feature values used in the simulations.

Phoneme	Power	Vocalic	Diffuse	Acute	Consonantal	Voiced	Burst	in McClelland and Elman (1986)
p	4	1	7	2	8	1	8	Yes
b	4	1	7	2	8	7	7	Yes
t	4	1	7	7	8	1	6	Yes
d	4	1	7	7	8	7	5	Yes
k	4	1	2	3	8	1	4	Yes
g	4	1	2	3	8	7	3	Yes
s	6	4	7	8	5	1	–	Yes
ʃ	6	4	6	4	5	1	–	Yes
r	7	7	1	2	3	8	–	Yes
l	7	7	2	4	3	9	–	Yes
a	8	8	2	1	1	8	–	Yes
i	8	8	8	8	1	8	–	Yes
u	8	8	6	2	1	8	–	Yes
ʌ	7	8	5	1	1	8	–	Yes
w	7	7	7	2	2	8	–	No
*℧*	8	8	7	3	1	8	–	No
f	6	4	7	3	5	1	–	No
ɒ	7	8	2	2	1	8	–	No
ə	7	8	4	1	1	8	–	No
I	8	8	7	6	1	8	–	No
ɑ	8	8	2	2	1	8	–	No
*θ*	6	4	7	4	5	1	–	No
n	7	6	7	7	4	8	–	No
m	7	6	7	2	4	8	–	No
ð	6	4	7	4	5	8	–	No
e	8	8	7	7	1	8	–	No
z	6	4	7	8	5	8	–	No
v	6	4	7	3	5	8	–	No
ʒ	6	4	6	4	5	8	–	No
j	7	7	8	8	2	8	–	No
*ε*	8	8	4	6	1	8	–	No
h	6	4	4	1	5	1	–	No
ŋ	7	6	2	3	4	8	–	No
ɔ	8	8	4	2	1	8	–	No

#### Sub-segmental detail

In order to further evaluate the generality of TRACE for studies of infant word recognition, the model is used to simulate results reported by White and Morgan (2008) who used an adaptation of the mispronunciation task. White and Morgan (2008) report a graded sensitivity to the severity of mispronunciations of familiar words and argue that lexical processing in toddlers is affected by sub-segmental phonological detail. We demonstrate that TRACE also displays a graded sensitivity to mispronunciation severity, but only if lexical or phonemic competition is suppressed when using infant lexicons with TRACE. Manipulation of these inhibitory parameters in TRACE has important implications for our understanding of the structure building processes in the developing lexicon. In particular, this result suggests that there is a developmental transition late in the second year of life from a lexicon structure without inhibitory processes to one where such processes play a central role in lexical recognition. We review some recent studies that argue for late onset of inhibitory processes in the infant lexicon and re-analyse some old data that are consistent with TRACE’s predictions.

#### Word learning and lexical competition

Finally, we use TRACE to implement Swingley and Aslin’s (2007) finding that words are learnt more easily when they belong to sparse phonological neighbourhoods than when they belong to dense neighbourhoods. TRACE successfully simulates their findings, despite the fact that it is not a model of word learning. Although this simulation does not rule out infant word learning in the original study, the manner in which TRACE succeeds suggests that Swingley and Aslin’s (2007) results can also be understood in terms of graded sensitivity to the mispronunciation of the object labels rather than as an indicator of novel word learning. Furthermore, this alternative interpretation of Swingley and Aslin’s (2007) findings is consistent with the changes in TRACE needed to simulate White and Morgan’s (2008) findings, namely, the manipulation of inhibitory competition parameters.

## Vowels and Consonants

### Comparing vowel and consonant mispronunciations

#### Background

Do vowels and consonants play a similar role in constraining lexical access in infant word recognition? Although it is uncontroversial that both vowels and consonants are critical for word recognition (*ball* vs. *bell* vs. *tell*), the relative importance of vowels and consonants in the phonological representations of early words has recently come under close scrutiny. For example, Nazzi (2005) describes a name-based, categorisation experiment, demonstrating that consonants are more discriminating than vowels in supporting lexical acquisition in 20-month-old French infants, confirming the view that lexical representations rely mainly on consonants (Nespor et al., 2003, Owren and Cardillo, 2006). Havy and Nazzi (2009) report similar findings in a word learning task with 16-month-old French infants, and Nazzi, Floccia, Moquet, and Butler (2009) argue that 30-month-old English and French infants “give less weight to vocalic information than to consonantal information in a lexically related task even though they are able to process fine vocalic information” (Nazzi et al., 2009, p. 522).

Other experimental tasks have demonstrated that infants are sensitive to minimal mispronunciations of onset consonants in familiar words from as young as 12-months (12 m: Mani and Plunkett, 2010b, Zesiger et al., 2012, 14 m: Swingley and Aslin, 2002, Ballem and Plunkett, 2005, 18–24 m: Bailey and Plunkett, 2002, Swingley and Aslin, 2000), and from 19 months of age when the medial consonant is changed (Swingley, 2003). Vowels mispronunciations of familiar words have not been studied as much as consonant mispronunciations until recently. Swingley and Aslin, 2000, Swingley and Aslin, 2002 suggest that vowels play a role in phonological representation from 14 months of age. However, this result was based on an analysis of vowel mispronunciations of just two words. Mani and Plunkett (2007) presented infants with medial vowel mispronunciations and correct pronunciations of familiar, monosyllabic CVC words at 15, 18 and 24 months of age. This detailed study (10 vowel mispronunciations at 15 months of age and 16 vowel mispronunciations at 18 and 24 months of age) confirmed Swingley and Aslin’s findings that infants are sensitive to vowel mispronunciations from as early as 15 months of age. In a follow-up study, Mani and Plunkett (2007, Experiment 2) compared directly infants’ sensitivity to vowel and consonant mispronunciations of the same familiar words. Infants detected both types of mispronunciations and showed no systematic differences in sensitivity to the two types of mispronunciation. More recently, Mani and Plunkett (2010b) have shown that 12 month-old infants can detect mispronunciations of medial vowels in familiar, mono-syllabic words. Mani and Plunkett (2007) argue for a symmetry in infants’ sensitivity to vowel and consonant mispronunciations of familiar words at 18 and 24 months of age, suggesting that both play an important role in constraining infant word recognition, a claim corroborated in a recent experiment with English 16- and 23-month olds (Floccia, Nazzi, Delle Luche, Poltrock, & Goslin, 2013).

Potential explanations of the divergent pattern of findings concerning the role of vowels and consonants in early lexical representations are varied. The tasks are different: Mani and Plunkett, 2007, Mani and Plunkett, 2010b, Swingley and Aslin, 2000, Swingley and Aslin, 2002 used an inter-modal preferential looking (IPL) task whereas Nazzi, 2005, Havy and Nazzi, 2009, Nazzi et al., 2009 used a name-based categorisation task. Different tasks may tap contrasting levels of sensitivity to vowel and consonant identity. The status of lexical items also differed in the two sets of studies (familiar words vs. novel words) and the studies were mostly conducted in different languages (English vs. French). Both lexical status and language identity may impact an individual’s sensitivity to variation in vowel and consonant pronunciations. Computational models like TRACE predict that sensitivity to lexical mispronunciations will be influenced by the competitive processes between lexical items (McClelland and Elman, 1986, Frauenfelder et al., 2001). For example, lexical entries from dense neighbourhoods should exhibit greater mispronunciation effects than those from sparse neighbourhoods. Since vocabulary structure and content is in a state of flux during early development, predictions regarding the impact of different types of mispronunciation are almost impossible to derive without detailed knowledge of early vocabularies and a tool for formal simulation. Following the successful comparison of TRACE with adults’ looking behaviour described in Allopenna et al. (1998), we investigate the impact of vowel and consonant mispronunciations using TRACE. More specifically, we attempt to mimic the looking behaviour of the infants studied by Mani and Plunkett, 2007, Swingley and Aslin, 2000 to gain insight into the source of any asymmetries between vowels and consonants that Nazzi and colleagues have reported for infant word recognition.

Multiple factors influence the efficiency of TRACE’s capacity to eliminate competing lexical candidates. We consider two important factors for which we can obtain empirical estimates based on analysis of infant data: First, the number of cohort and neighbourhood competitors and second, the token frequency of individual word candidates. A large number of competitors delays the identification of a successful candidate in TRACE whereas high token frequency speeds identification. The model eliminates the noise of infant performance introduced by inattention, memory failure and variability of individual lexicons and permits a precise evaluation of the impact of the phono-lexical processes inherent in TRACE for infant word recognition. Furthermore, the model allows us to manipulate vocabulary size in a manner that mimics lexical development during infancy, thereby permitting an evaluation of the potential impact of the size and structure of infant lexicons on their sensitivity to the mispronunciations of familiar words. We will show how TRACE supports Mani and Plunkett’s (2007) claim that both vowels and consonants constrain lexical access to familiar words in the infant lexicon and demonstrate how this simulation accommodates Swingley and Aslin, 2000, Swingley and Aslin, 2002 mispronunciation studies with 18–24 and 14-month old infants. However, TRACE also predicts that infants should become increasingly sensitive to onset mispronunciations (usually consonants in English) of familiar words as vocabulary develops, whereas their sensitivity to non-onset (often vowels) mispronunciations should remain relatively stable during the second year of life. We also demonstrate that TRACE shows greater sensitivity to onset consonant mispronunciations in the absence of a competing distracter, pointing to the impact of task-specific factors when assessing the role of vowel and consonants in infant word recognition. Furthermore, TRACE simulations show that high frequency words exhibit a greater asymmetry between consonant and vowel mispronunciations than low frequency words. These findings are consistent with recent reports of asymmetries in sensitivity to vowel and consonant mispronunciations during later stages of vocabulary development (Nazzi et al., 2009).

It is important to note that TRACE is not itself a developmental model. All developmental trends emerging from the simulations can be attributed exclusively to lexical competition arising from changes introduced to the size and structure of TRACE’s ‘mental’ lexicon. It is also important to note that TRACE makes no special distinction between vowels and consonants, neither in the original model nor in our adaptation.^1^ In a first simulation, we evaluate the accuracy of TRACE in capturing the impact of vowel and consonant mispronunciations on recognition of familiar words as reported by Mani and Plunkett (2007). Mani and Plunkett (2007) used an IPL task (Golinkoff, Hirsh-Pasek, Cauley, & Gordon, 1987) in which target and distracter objects are presented side-by-side on a computer monitor while the target object is named over a loudspeaker using either a correct pronunciation or a mispronunciation. Preference for the target object was used as an index of the infant’s sensitivity to an association between the heard label and the visual object. In the simulation, we compare looking times to the target when its label is pronounced correctly, when the medial vowel is changed and when the onset consonant is changed. Looking time measures are simulated in an analogous fashion to Allopenna et al. (1998), using an implementation of Luce’s forced choice rule.

It might be argued that TRACE is not the appropriate computational framework for simulating visual world tasks like IPL as previous research with infants assumes that object identification in a visual world task proceeds from hearing the name to generating a semantic representation which is then matched to the visual image (Swingley & Fernald, 2002). TRACE possesses no semantic nor visual representations, so no direct matching to the visual image is possible. However, adults are capable of implicit generation of the names of visually fixated images (Meyer, Belke, Telling, & Humphreys, 2007). A phono-lexical matching process between the heard label and the label for the perceived object can then determine the goodness-of-fit. A similar linking hypothesis can operate for infants. Recent research has demonstrated that infants can and do generate implicit labels for name-known objects when these objects are viewed in the context of a preferential looking task (Mani & Plunkett, 2010a). Therefore, we adopt a similar strategy to Allopenna et al. (1998) in assuming that target preferences in the infant IPL task can be simulated by identifying TRACE’s best match to the phono-lexical representations of the names associated with the target and distracter objects.

#### Method

We used jTRACE (Strauss et al., 2007), a re-implementation of the TRACE model (McClelland & Elman, 1986) to simulate Experiment 2 of Mani and Plunkett (2007). We created typical lexicons for 15, 18 and 24 month olds by compiling Oxford CDIs (Hamilton, Plunkett, & Schafer, 2000, a UK adaptation of the MacArthur-Bates CDI, Fenson et al., 1993) using words that are understood by at least 50% of the infants in each age group.^2^ The lexicons are specified using data from 50 infants at 15 months, 179 infants at 18 months and 81 infants at 24 months of age and include 49, 131 and 217 words, respectively. Whereas all stimuli used by Mani and Plunkett (2007) belong to the typical lexicons of 18 and 24 month old infants, *bib, bike, boot, bus, coat* and *keys* were added to the 15-month-old TRACE lexicon, even though they are only known, according to the Oxford CDI (Hamilton et al., 2000), by less than 50% of the infants at that age. This modification permitted a replication of Mani and Plunkett (2007) using their whole stimulus set. A complete lexicon listing for each age group is provided in Appendix B.

Recognition time for spoken words is affected not only by the number of phonological neighbours (Miller & Eimas, 1995), but also by their frequency (Goldinger et al., 1989). We identified individual token frequencies, by extracting word frequencies on all tiers based on the Manchester corpora (Theakston, Lieven, Pine, & Rowland, 2001) from the CHILDES database (MacWhinney, 1991), where 12 English children were recorded weekly from 20 to 36 months of age. Word frequencies used in the simulations are raw word counts on the whole corpora, converted to frequency per million. We compare simulations obtained when word frequency is allowed to vary in the model with simulations when frequency is kept constant, so as to identify the contributing roles of frequency and lexicon size, independently. When implementing frequencies in the model, we follow the practise advocated by MacKay (1982) and implemented by Dahan, Magnuson, and Tanenhaus (2001a), i.e., frequency modulates the connection weights associated with lexical units, using the same value for the scaling parameter (0.13) used in Dahan et al. (2001a). The modulation of frequency effects via phoneme-lexicon connection strengths is consistent with a Hebbian style, frequency-based learning mechanism. Dahan et al. (2001a) found this type of bottom-up connection strength implementation to have qualitative advantages over resting state and post-perceptual frequency manipulations. The control simulations used the default model, as introduced by McClelland and Elman (1986), where frequencies were not allowed to vary.

Given the large size of the infant lexicon at 24 months of age, many of the phonemes needed to represent the different words were not encoded in the original TRACE model (McClelland & Elman, 1986) nor in its re-implementation (Strauss et al., 2007). Therefore, we added feature values for all phonemes used in the infant’s lexicon.^3^ Table 1 reproduces the feature value of all phonemes used in the simulations. Long vowels such as uː, iː, əː, ɔː, *ε*ː, ɑː are implemented as being twice as long as their respective short counterparts u, i, ə, ɔ, *ε*, ɑ, while keeping the same feature values. Note that if some of the original phonemes used in McClelland and Elman (1986) take more than one value within a feature, all new phonemes possess a unique value on each feature, as reported in Table 1. All words in the lexicon were encoded using the IPA phonemes listed in Table 1, using southern British English pronunciation. Vowel and consonant mispronunciations were encoded using the phonetic description reported in Mani and Plunkett (2007), reproduced in Table 2.

**Table 2:** Correctly pronounced and mispronounced labels presented to infants in Experiment 2 of Mani and Plunkett (2007). Note that targets and distracters have the same onset consonants, so that onset consonants alone cannot be used to identify the target.

Correct pronunciations	Mispronunciations		Distracter
	Vowel	Consonant	
Ball/bɔːl/	Bule/buːl/	Gall /gɔːl/	Bear/b*ε*ː/
Bib/bIb/	Bab/bab/	Dib/dIb/	Boot/bu:t/
Bed/b*ε*d/	Bud/bʌd/	Ped/p*ε*d/	Book /b*℧*k/
Bus/bʌs/	Bas/bas/	Pus/pʌs/	Bike /bʌIk/
Cat/kat/	Cart/kɑt/	Gat/gat/	Cow/ka*℧*/
Cup/kʌp/	Cep/k*ε*p/	Gup/gʌp/	Car /kɑː/
Dog/dɒg/	Doog/d*℧*g/	Bog/bɒg/	Duck /dʌk/
Keys/kiːz/	Kas/kaːz/	Tees/tiːz/	Coat /kə*℧*t/

Correctly pronounced words and mispronounced words are presented to the model and activation levels of two competitors (the target and a distracter) are monitored. We adopt the same linking hypothesis as Allopenna et al. (1998) in order to map the activation levels to fixation durations. Activation of a word is the result of both its direct activation due to phonological overlap with the input and the result of competition with all other words that are activated with that same input. Only items that are on display are available as potential responses. The activation levels *a* of the displayed items are then transformed into response strengths following Luce (1959). Given the high salience of the images, we assume that total looking time is split entirely between the target and distractor objects, enabling us to convert the response strengths into fixation durations using the Luce choice rule. The proportion of looking to the target at time *t* is given by:

(1) $$p_{\mathrm{target}}(t)=\frac{e^{{\mathrm{ka}}_{\mathrm{target}}(t)}}{e^{{\mathrm{ka}}_{\mathrm{target}}(t)}+e^{{\mathrm{ka}}_{\mathrm{distractor}}(t)}}$$

where *k* is a free parameter determining the amount of separation between units of different activations. We set k=2, as opposed to a higher degree of separation, k=4, typically used for adults. The lower value for *k* accounts for the fact that infants tend not to focus during a whole trial on a single image even in the case of a highly familiar object. All other parameters used in TRACE were set to their default values. Proportion of looking times to the targets and distracters are reported as the average over 100 processing cycles starting with the onset of the pronounced word. An approximate mapping between experimental data and simulations is described later, yielding an estimate of about 20 ms per simulation cycle. One hundred simulation cycles thus correspond to about 2000 ms, which closely approximates the duration of the post-naming phase used by Mani and Plunkett (2007).^4^ Table 2 presents the stimuli used in the original experiment and in the current simulation.

#### Results

**Fig. 1:**
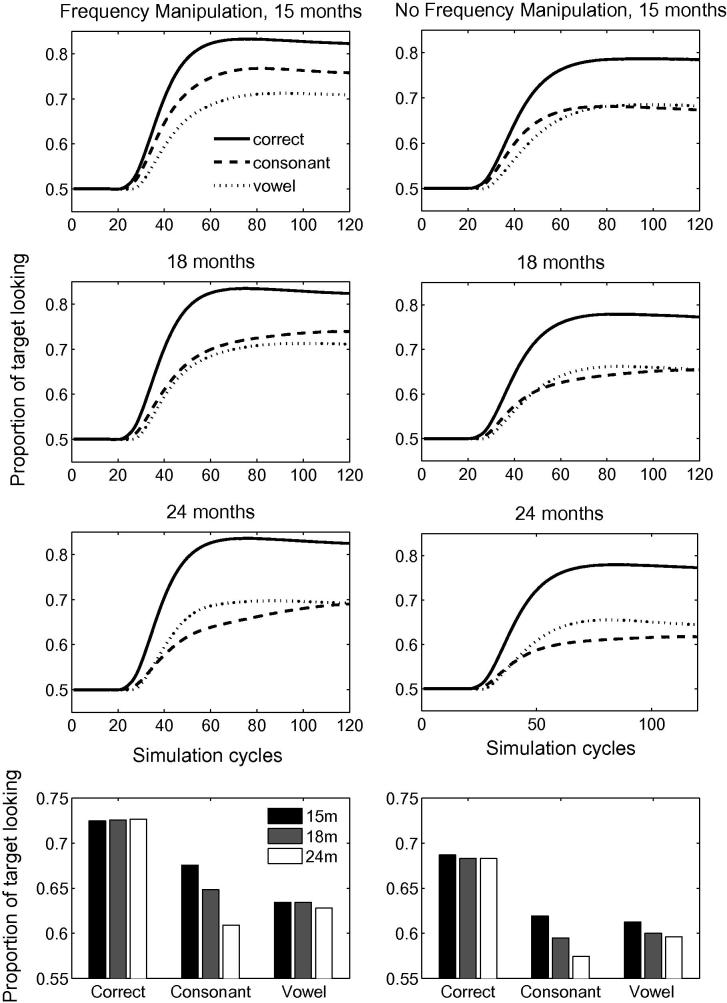
Time course of proportional looking in TRACE associated with correct pronunciations and both consonant and vowel mispronunciation for 15-, 18- and 24-month lexicons. The right-hand panels depict activation levels for items where frequency is held constant. The left-hand panels depict frequency modulated activation levels for the same items. Bottom panels indicate overall target looking for pronunciation types at each lexicon size.

Fig. 1 depicts the time course of activation levels in TRACE of the target items for correct pronunciations and mispronunciations used by Mani and Plunkett (2007), both when word frequency is held constant across vocabulary items (right panels) and when frequency information is incorporated into the model (left panels). Raw activation levels are converted to proportional target looking measures according to Eq. (1). For all lexicon sizes, TRACE achieves a high level of proportional target looking for correct pronunciations (≈80% where chance looking is 50%) within 40 cycles of label onset. TRACE accurately identifies the target. A reduction in proportional looking time at the target is observed for both consonant (≈60% to 75%) and vowel mispronunciations. The impact of lexicon size and frequency manipulation on the proportion of target looking is greater for consonant than vowel mispronunciations.

**Fig. 2:**
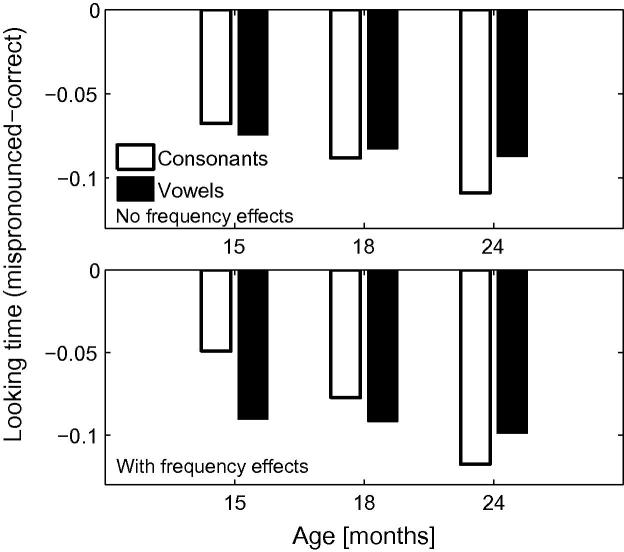
Mispronunciation sensitivity (differences between the correct and incorrect pronunciations) as obtained by the model for the 15-, 18- and 24-month lexicons, for vowel and consonant mispronunciations. Note the developmental trend for consonant mispronunciations.

To highlight the impact of lexicon size and frequency on mispronunciations, Fig. 2 depicts mispronunciation sensitivity as expressed as the difference in looking times between the correct and incorrect pronunciations for the lexicon sizes corresponding to the 3 age groups, for both frequency conditions. It is apparent from Fig. 2 that TRACE readily discriminates between correct and mispronunciations both when the mispronunciation involves a vowel or a consonant. Looking times at the target were longer for correct pronunciations compared to mispronunciations, verifying the contributing role of vowels and consonants in constraining auditory word recognition in TRACE when using representative infant lexicons, thereby aligning with Mani and Plunkett’s (2007) claim that infants are sensitive to vowel and consonant mispronunciations as early as 15 months of age. The effect holds when word frequency is manipulated or held constant. Introducing variations of word frequency in the model increased overall looking times at targets in all pronunciation conditions. TRACE looked longer at the target for high frequency words.

Fig. 2 also indicates that TRACE’s sensitivity to vowel mispronunciations is quite stable across lexicon size whereas sensitivity to consonant mispronunciations increases with lexicon size. Note that, in the model, the only factor that varies with age is the size of the lexicon itself. This finding predicts that infants should become increasingly sensitive to consonant mispronunciations as their lexicon grows in size. No such change in sensitivity is predicted for vowel mispronunciations over the lexicon range explored in this model. Similar findings were obtained for the simulations with and without frequency variations, indicating that the increasing sensitivity to consonant mispronunciations is driven by the size of the lexicon rather than being an artifact of the relative token frequencies of individual lexical items, though the *rate* of increase in sensitivity to consonant mispronunciations is accentuated by incorporating frequency information.

**Fig. 3:**
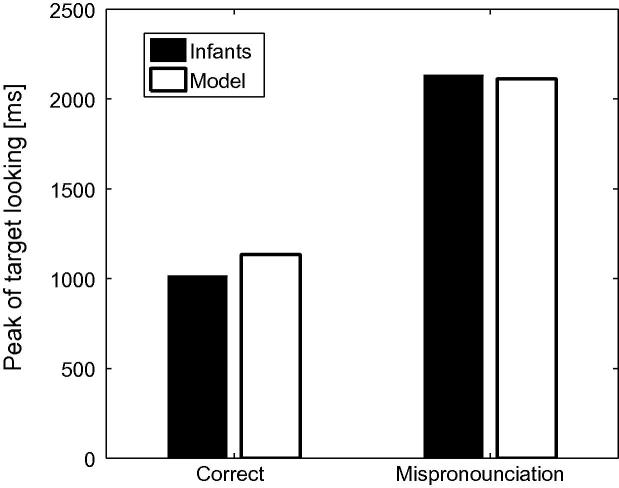
Peak times of target looking for correct pronunciation and mispronunciations, for the infants data obtained in Mani and Plunkett (2007) and for the model. Target looking for mispronunciations peaks much later than for correct pronunciations suggesting that, potentially, longer trial duration would have revealed a naming effect for mispronunciations too.

It is noteworthy that an effect of naming was observed for mispronunciations as well as correct pronunciations and for all lexicon sizes.^5^ Mispronunciation naming effects were not found in Mani and Plunkett (2007). In order to identify the locus of this discrepancy, we re-analysed the original infant data to identify the time at which the probability of fixating the target is highest, averaged across all participants, for both correct pronunciations and mispronunciations. Fig. 3 depicts the time in the naming phase at which target looking peaks for both correct pronunciations and mispronunciations. Target looking peaked 1013 ms into the naming phase for correct pronunciations and at an average of 2133 ms for mispronunciations, which corresponds to the end of the trial. This finding suggests that, potentially, a longer trial duration would have revealed a naming effect in the case of mispronunciations.

In order to compare infant behaviour with model performance, we determined the number of simulation cycles required to achieve peak target looking in the correct pronunciation condition. Alignment of TRACE’s peak looking after word onset with infant peak looking times for correct pronunciations indicated that each simulation cycle corresponded to 20 ms^6^ of infant looking time (allowing 367 ms needed to elicit language mediated eye-movements not required by TRACE). Peak looking time for mispronunciations in the simulation was then extrapolated to predict the peak looking time in response to mispronunciations for infants. Fig. 3 depicts the peaks of target looking as obtained by the model, using cycle durations of 20 ms, for both the correct pronunciations (1133 ms) and the mispronunciations (2113 ms). The model predicts correctly that looking times at the target should peak much later for mispronunciations than for correct pronunciations, again suggesting that a longer trial duration may have revealed a naming effect.

**Fig. 4:**
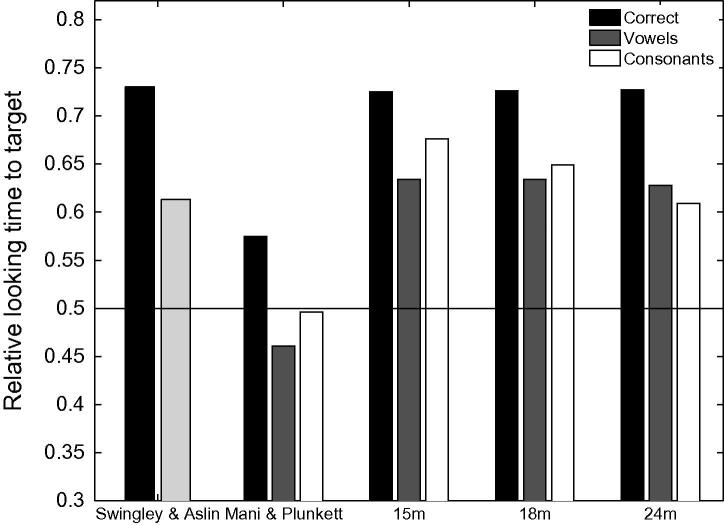
Target preferences for correct pronunciations, vowel mispronunciations and consonant mispronunciations in TRACE with 15-, 18- and 24-month old lexicons. Note the close agreement of the TRACE simulations with Swingley and Aslin’s (2000) data for 18–23 month olds where all mispronunciations are aggregated (light grey). For comparison, looking preferences, aggregated over the age range, for correct pronunciations and for vowel and consonant mispronunciations in Mani and Plunkett (2007) are also reported.

Longer trials were used by Swingley and Aslin (2000) as trials ended 6 s after the onset of the sentence (allowing for a carrier phrase of about 765 ms, this leaves more than 5 s post-naming), as opposed to 2.5 s after the onset of the target name in Mani and Plunkett (2007). Swingley and Aslin (2000) reported a mispronunciation naming effect for infants between 18 and 23 months of age (73% of looking time at target for correctly pronounced labels and 61.3% for mispronunciations). A naming effect for mispronunciations was also reported by Bailey and Plunkett, 2002, Ballem and Plunkett, 2005. Fig. 4 provides a graphical comparison of Swingley and Aslin, 2000, Mani and Plunkett, 2007 data to the simulation results. The mispronunciations reported in Swingley and Aslin, 2000 correspond to the aggregation of four consonant mispronunciations and two vowel mispronunciations.

The close agreement between the simulation results and Swingley and Aslin’s (2000) experimental data show that, like the infants, TRACE can identify the target referent in a forced choice task when the target is mispronounced. TRACE succeeds over a broad range of mispronunciation types and vocabulary sizes even when the name of the distracter has the same consonant onset as the target (as in Mani & Plunkett, 2007). Note that this constraint did not hold in the Swingley and Aslin (2000) study where targets and distracters had different onsets, suggesting that overall phonological neighbours as well as cohort competitors have an impact on target looking in these inter-modal preferential looking studies.

**Fig. 5:**
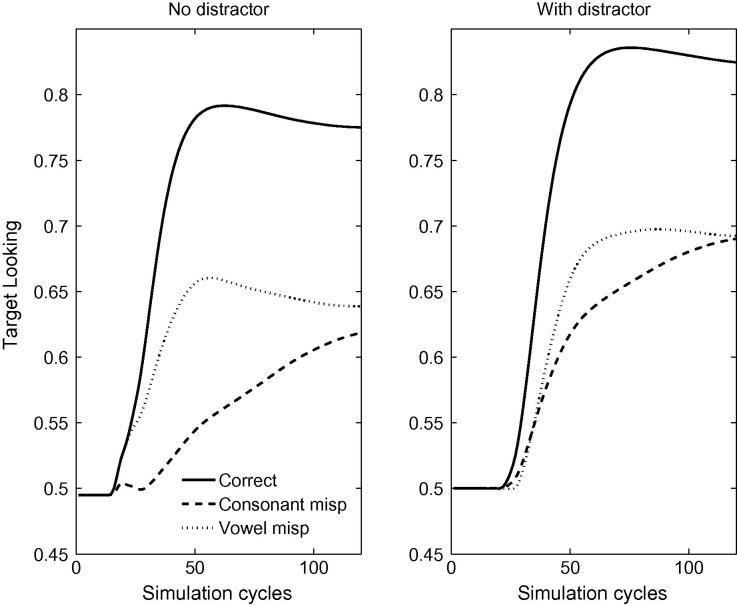
Time courses of activation levels of the target items for the correct, the onset-vowel and the medial-consonant mispronunciations for the 24-month artificial lexicon in the presence or absence of a distracter.

It is possible to relax the assumption that the distractor object competes with the target object for attention by setting the distractor label activation to zero when applying the Luce choice rule. This manipulation is equivalent to changing the task characteristics so that TRACE only evaluates the goodness-of-fit of the auditory signal (correct or mispronunciation) to the target item in the context of existing lexical knowledge. Fig. 5 reveals that in the absence of a competing distractor, TRACE exhibits increased early sensitivity to onset consonant mispronunciations compared to the case when the distracter is present. In contrast, TRACE’s sensitivity to vowel mispronunciations is little affected by the presence or absence of a distractor. An implication of this finding is that tasks involving a distractor whose onset is shared with the target *diminish* any asymmetry in sensitivity to vowel and consonant mispronunciations whereas distractor absent tasks *enhance* the asymmetry.

#### Discussion

A comparison of simulation results to experimental data reported by Mani and Plunkett, 2007, Swingley and Aslin, 2000 suggests that the TRACE model, when supplemented with the Luce choice rule, appropriately captures infants’ looking preferences in a forced choice interpretation of the IPL task. The model, as with the infants, showed greater target looking when the target is correctly pronounced than when it is mispronounced. Looking times at the target in the model were reduced for both onset consonant changes *and* medial vowel changes for all lexicon sizes, mimicking infants’ sensitivities to vowel and consonant mispronunciations in familiar words present from an early age (Mani and Plunkett, 2007, Mani and Plunkett, 2010b). However, the simulations predict that infant sensitivity to vowel and consonant mispronunciations should exhibit different developmental trajectories; while lexicon size has little impact on target preferences for correct pronunciations and vowel mispronunciations, a lexicon size effect is predicted for consonant mispronunciations: Sensitivity to consonant mispronunciations increases with lexicon size. This result is obtained when word frequency is held constant in the model—a finding consistent with developmental accounts that attribute discriminability between words to neighbourhood density (Jusczyk, 1993). However, that the rate of increase in sensitivity to consonant mispronunciations with lexicon size is enhanced by incorporating frequency information into the model, points to a role for experience with individual words, a position consistent with the familiarity hypothesis (Metsala, 1999). The increasing sensitivity to consonant mispronunciations of familiar words when competition from a distractor is eliminated might also help explain the preferential attention to consonant over vowel information in word learning tasks reported by Nazzi, 2005, Nazzi et al., 2009 in 20- and 30-month old infants. It is difficult to judge whether the name-based categorisation task employed by Nazzi and colleagues differs in this respect from standard mispronunciation tasks (e.g., Mani and Plunkett, 2007, Swingley and Aslin, 2000). However, insofar as tactile manipulation of an object focuses infant attention entirely upon that object, then distracters might be deemed absent in name-based categorisation.

Inconsistencies in the observation of naming effects induced by mispronunciations across several experimental studies (Bailey and Plunkett, 2002, Ballem and Plunkett, 2005, Mani and Plunkett, 2007, Swingley and Aslin, 2000) are readily resolved in the TRACE framework. TRACE predicts that peak target looking for mispronunciations is achieved later than for correct pronunciations. Failure to observe mispronunciation naming effects in some studies (e.g., Mani & Plunkett, 2007) can be attributed to the short picture display times used.

In TRACE, competition occurs at the levels of phonemes and words. The model contains no semantic associations nor semantic representations of any kind. In this account, upon hearing a word, its phonological content is compared to the phonological content associated with both pictures presented to the infant; the target and the distractor. Words in infants’ lexicons compete at the level of their *phonological* overlap. Although semantic information may be accessed shortly after hearing the word, as suggested by Swingley and Fernald (2002), only the phonological locus of the mismatch needs to be identified in order to explain the effects of vowel and consonant mispronunciations and the naming effect observed in infants. This finding is consistent with recent evidence showing that infants can implicitly generate a phonological representation for the name of a currently fixated object, which can in turn influence their looking preferences when they hear a name (Mani & Plunkett, 2010a). As a consequence, the patterns of findings observed in studies such as Mani and Plunkett, 2007, Swingley and Aslin, 2000 can be explained computationally in terms of phonological competition alone.

### Cohort competition effects

In the previous simulations, age groups are modelled by installing their typical lexicons in TRACE. Word frequencies, phonological features and all other parameters are kept constant across lexicons. Therefore, lexicon size effects (or age effects) in the model are driven solely by the differing sets of competitors (the infant’s lexicon) at 15, 18 and 24 months of age. The initial portion of the word is important for activating potential lexical candidates as proposed by the Cohort account (Marlsen-Wilson & Welsh, 1978). As the size of the lexicon increases, the set of competitors increases, thereby impacting directly the set of cohort competitors. For these relatively small lexicons, the probability that any lexical entry has a phonological neighbour remains quite small. Table 3 displays the number of cohort competitors for different onset consonants used in Experiment 2 of Mani and Plunkett (2007) in the typical lexicon at 15, 18 and 24 months of age as estimated from the Oxford CDI (Hamilton et al., 2000). The number of potential lexical competitors increases dramatically in this age range. For example, the number of *t*-onset words increases by a factor 5 in the three month period from 15 to 18 months, *p*-onset words increase by a factor 20 between 15 and 24 months and the first *g*-onset words appear at 18 months.

**Table 3:** Number of cohort competitors for the different onset consonants used in Experiment 2 of Mani and Plunkett (2007) at 15, 18 and 24 months of age. The mean cohort size per onset phoneme is also reported and confirms the increase in competition with age.

Onset phoneme	15 m	18 m	24 m
b	15	31	37
d	5	7	10
g	0	3	7
k	7	12	17
p	1	5	20
t	4	21	24

Mean	4.08	5.95	9.04

#### Phoneme boundaries

**Fig. 6:**
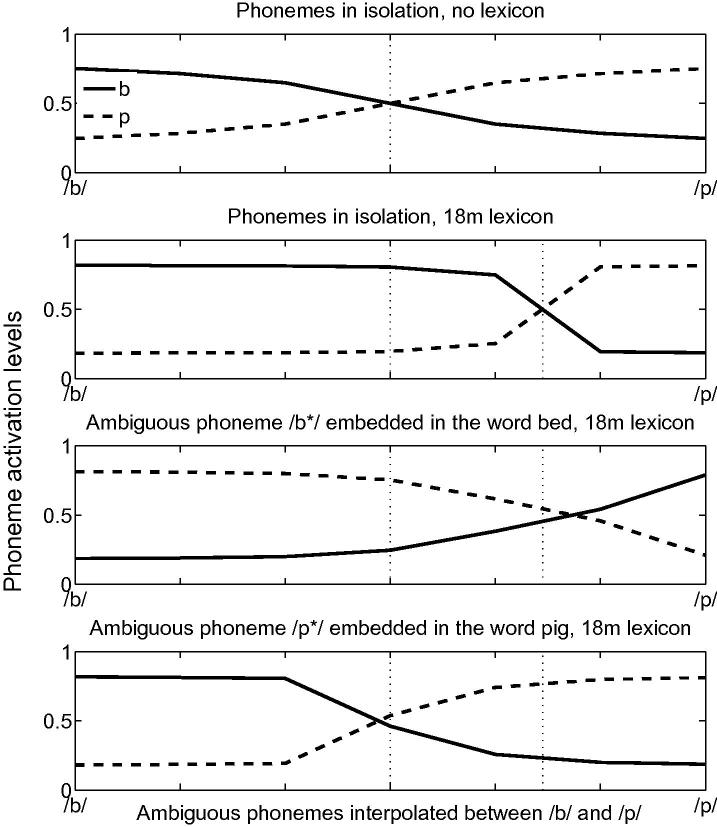
Top panel: In the absence of lexical influences (no feedback from the lexical level to the phoneme level), ambiguous phonemes half-way between /b/ and /p/ activate both phonemes equally. Second panel: When phono-lexical connections are present, the excess of b-onset words in a 18-month lexicon induces a shift so that more ambiguous phonemes are identified as /b/ phonemes. Third panel: When the ambiguous phonemes are embedded in a word starting with /b/, the shift is maintained. Fourth panel: When the ambiguous phonemes are embedded in a /p/-onset word, lexical influences compensate the asymmetry due to the excess of /b/-onset words in the lexicon.

In the model, the dramatic increase in lexical size around the time of the vocabulary spurt (18–21 months) impacts directly phonological sensitivity to onset consonant mispronunciations. By way of illustration, we compared onset consonant mispronunciations of words belonging to large cohorts (b-onset words) to mispronunciations of words from small cohorts (p-onset words). Any asymmetries deriving from cohort size would further establish the role of lexical structure on speech perception and sensitivity to mispronunciations in TRACE and, by implication, offer predictions for infant sensitivity to mispronunciations. All simulations were conducted with the 18-month lexicon. A continuum between /b/ and /p/ was created by interpolating feature values of both phonemes. The top two panels of Fig. 6 display phoneme activation levels in TRACE when ambiguous phonemes along the /b/–/p/ continuum are presented in isolation, with or without,^7^ lexicon-phonemic feedback connections, respectively. The top panel of Fig. 6 shows that phonemes half-way between /b/ and /p/ activate both phonemes equally well in the absence of lexical feedback, and that ambiguous phonemes closer to their prototypes are disambiguated well. In the presence of lexico-phonemic connections (second panel of Fig. 6), a substantial proportion of the ambiguous phonemes closer to the prototype /p/ are recognised as /b/ phonemes. The dominance of /b/-onset words in the 18-month-old lexicon leads to a higher activation level of /b/ phonemes at the phoneme level, resulting in a shift of the perceptual boundary between /b/ and /p/.

The third and fourth panels of Fig. 6 depict phoneme activations in TRACE when the same continuum is substituted for the initial phoneme in an existing entry in the 18-month-old lexicon, with lexical-phonemic feedback present: [$b^{*}\mathrm{ed}$] or [$p^{*}\mathrm{ig}$], the asterisk designating the ambiguous phoneme between /b/ and /p/. The shift in perceptual boundary is small when the ambiguous phoneme is part of [$b^{*}\mathrm{ed}$], primarily due to saturation effects (a non-ambiguous input activates the corresponding phoneme even when lexical influences are present — see Appendix A). In contrast, when the ambiguous phoneme is part of [$p^{*}\mathrm{ig}$], the lexical effect is strong enough to counteract the impact of an unbalanced lexicon at 18-months where more words start with /b/ than /p/. These simulations highlight the potential impact of the structure of the infant lexicon on phoneme perception, when viewed from the theoretical framework offered by TRACE.

#### Lexical effects

**Fig. 7:**
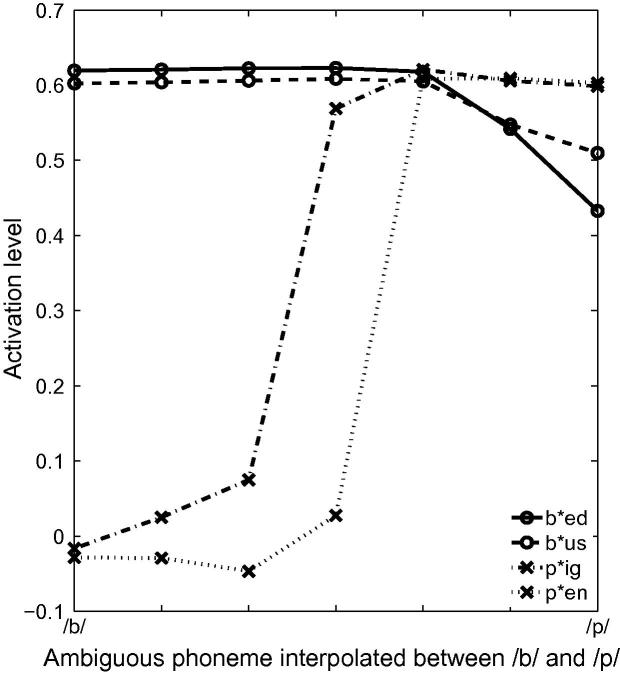
Mispronunciations of /b/-onset words lead to slightly reduced activation associated with the target words (bed and bus). In contrast, when /p/-onset words are mispronounced, word recognition is impaired. The source of this asymmetry lies in the imbalance of /b/- to /p/-onset words in infant lexicons.

Next, we consider the impact of these structural asymmetries at the lexical level by quantifying TRACE’s sensitivity to mispronunciations of words taken from large cohorts and small cohorts. The initial consonants of two /b/-onset words (*bed* and *bus*) and two /p/-onset words (*pig* and *pen*) were substituted with tokens taken from the same /b/–/p/ continuum used before. Fig. 7 displays the activation levels associated with these four words embedded with the ambiguous tokens from this continuum. The activation of /b/-onset words is only minimally affected by mispronunciations of the onset consonant /b/, such that even one-feature mispronunciations (a /p/ instead of /b/) result only in minor reductions in the levels of activation of the /b/-onset words. In contrast, /p/-onset words are more sensitive to mispronunciations and when /p/-onset words are mispronounced as /b/, the mispronunciations are not recognised.

#### Summary of cohort competition effects

These results suggest further empirical predictions for infant speech perception and word recognition: First, the size and structure of infant vocabularies should have an impact on the location of perceptual boundaries between related phonemes. The perceptual boundary for the /p/–/b/ contrast should shift such that ambiguous phonemes close to this boundary are assimilated to the phonemic category corresponding to the phonemic segment which has the larger cohort in the infant lexicon—in this case, the phoneme /b/.

In contrast to adult speech perception where perceptual boundaries between phonemes are assumed to remain unaffected by minor changes/additions to vocabulary, TRACE predicts that the perceptual boundaries between phonemes will shift as vocabulary is acquired during infancy. These shifts are most likely to occur during periods of dramatic vocabulary development, such as the vocabulary spurt. Second, infant sensitivity to mispronunciations of lexical items should also be conditioned by the structure and content of their lexicons. In particular, we predict an asymmetry in sensitivity to mispronunciations of word-initial phonemes, when the two phonemes are phonetically related but define lexical cohorts that differ in size. Thus, infants should more readily tolerate a /b/-onset word mispronounced with a /p/ as a token of the intended word, than they will tolerate a /p/-onset word mispronounced with a /b/. The degree of tolerance demonstrated by infants in this regard will change as the structure and content of their lexicon changes.

These results indicate that infant lexical development can offer a unique perspective into interactions between lexical effects and phoneme perception. Early vocabularies contain fewer words and parts of lexical space are less densely populated than others, resulting in a marked imbalance in cohort sizes. Consequently, lexico-phonetic effects are likely to be more salient with an infant vocabulary than with an adult one. Development may magnify behavioural effects that are otherwise more subtle in the adult system where the role of top-down lexico-phonemic connectivity has been hotly disputed for decades (Massaro, 1989, McClelland, 1991, Norris et al., 2000, Norris et al., 2003, Samuel, 1997).

In contrast, sensitivity to medial vowel mispronunciations is unaffected by the size of the lexicon, over the age range considered. A correct pronunciation for the onset of a word already reduces dramatically the set of potential candidates competing for recognition. The network dynamics for TRACE operates differently for medial vowel mispronunciations compared to the situation where many words are activated after the wrong phoneme is used for an onset. Hence, vowel mispronunciation effects are relatively insensitive to changes in vocabulary size.

### Positional effects

In order to disentangle the roles of the location of the mispronunciation (onset or non-onset) and the type of mispronunciation (vowel or consonant), we created artificial lexicons by inverting the first (onset) and the second phonemes in each word of the 15-, 18- and 24-month lexicons. By doing so, the artificial lexicons become rich in vowel-onset words while maintaining features that are known to affect speech perception, such as word length or word frequency. The mispronunciation constraints introduced by Mani and Plunkett (2007) are maintained as closely as possible: the distracters share the same onset as the target, and the same magnitude of mispronunciations are used. However, onset mispronunciations are now vowel mispronunciations and non-onset mispronunciations are consonant mispronunciations. Table 4 displays the stimuli used in these simulations.

**Table 4:** Correctly pronounced and mispronounced labels simulated with artificial lexicons obtained by exchanging the first and the second phonemes in each word. Note that targets and distracters have the same onset vowels.

Correct pronunciations	Mispronunciations		Distracter
	Vowel	Consonant	
/ɔːbl/	/uːbl/	/ɔːgl/	/ɔːhs/
/Ibb/	/abb/	/Idb/	/Ipg/
/*ε*bd/	/ʌbd/	/*ε*pd/	/*ε*tdib*ε*/
/ʌbs/	/abs/	/ʌps/	/ʌbIk/
/akt/	/ɑkt/	/agt/	/ak*℧*/
/ʌkp/	/*ε*kp/	/ʌgp/	/ʌdk/
/ɔdg/	/*℧*dg/	/ɔbg/	/ɔsk/
/iːks/	/aːks/	/iːts/	/iːʃp/

**Fig. 8:**
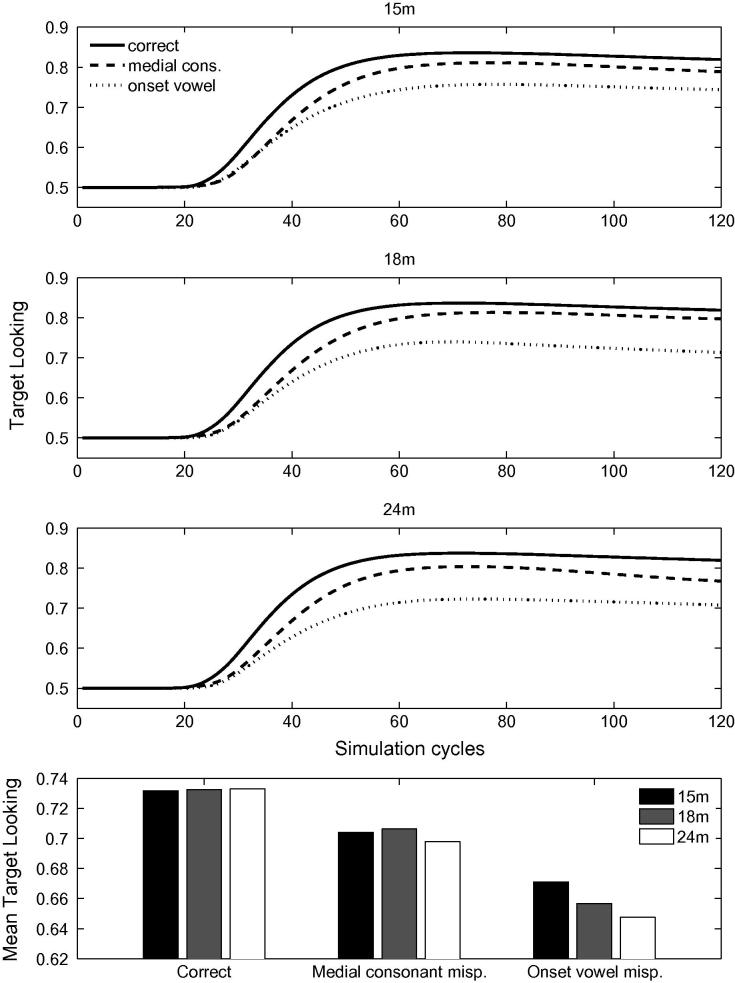
Time courses of activation levels of the target items for the correct, the onset-vowel and the medial-consonant mispronunciations, for the 15-month (top panel), the 18-month (second panel) and the 24-month artificial lexicon (third panel). The fourth panel displays the mean looking times towards the target, averaged over 100 processing cycles.

Fig. 8 displays the activation levels of the target items for correct, onset-vowel and medial-consonant mispronunciations, for the 15-month (top panel), the 18-month (second panel) and the 24-month artificial lexicons (third panel). The fourth panel of Fig. 8 displays the mean looking times towards the target, averaged over 100 processing cycles. As in the simulation of Mani and Plunkett (2007), vowel and consonant mispronunciations affect looking times: both mispronunciation types yield reduced looking compared to correct pronunciations. Mispronounced targets are also recognised, as looking times towards the targets always exceed 0.5. As with the earlier set of simulations, *medial* mispronunciations were largely unaffected by vocabulary size and structure whereas *onset* mispronunciations exhibited a developmental trend. However, the role of vowels and consonants is now reversed. Sensitivity to onset *vowel* mispronunciations increases with vocabulary size whereas sensitivity to medial *consonant* mispronunciations does not. This reversal is achieved despite the fact that consonants remain more contrastive than vowels, in the sense that there are a greater range of consonants in the lexicon than vowels.

This set of simulations with an artificial lexicon demonstrates that the structure of the lexicon impacts the perception of onset mispronunciations to a greater extent than the perception of non-onset mispronunciations. TRACE predicts that segment *location* plays a more decisive role in word recognition than segment *type* (vowel or consonant).

#### Summary

Increasing sensitivity to onset consonant mispronunciations in TRACE is directly influenced by the number of cohort competitors, a by-product of the increasing size of the lexicon whereas sensitivity to medial vowel mispronunciations is not. Absence of increased sensitivity to non-onset changes need not be attributed to the fact that it is a vowel change. Any change to non-onset phonemes should have a similar impact. This prediction is supported by findings that infants are also sensitive to medial consonant mispronunciations (Swingley, 2003). As discussed above, the number of cohort competitors directly impacts the sensitivity to onset mispronunciations. Therefore, we predict that a language with a substantial incidence of onset vowel words should display a strong sensitivity to onset vowel mispronunciations which increases with age.

## Sensitivity to Sub-segmental detail

Infants show graded sensitivity to mispronunciations of familiar words as a function of the severity of the mispronunciation. White and Morgan (2008) report that 19-month-olds show a graded response in their looking behaviour to a target picture when presented with a correct pronunciation, 1-feature, 2-feature or 3-feature mispronunciation of the onset consonant of a target word: Infants look longer at the target object when supplied with more accurate renditions of the target object’s name. In their experiment, the two pictures corresponded to a familiar target object and a novel object. In contrast to other mispronunciation experiments (e.g., Mani and Plunkett, 2007, Swingley and Aslin, 2000), the distracter image is name-unknown and does not represent a potential competing lexical entry. White and Morgan (2008) argued that using a novel object as a distracter is important for demonstrating graded sensitivity as it offers the infant the opportunity to consider the mispronunciation as a label for the novel distracter. This possibility is not available to the infant when the distracter is a name-known object. Thus, Bailey and Plunkett (2002) failed to find a systematic graded effect of mispronunciation at 18–24 months: Their experimental design differed to the one described in White and Morgan (2008) insofar as the distracter was also a familiar object, thereby offering infants with a potential lexical competitor. On the basis of their experimental findings, White and Morgan (2008) argued that lexical processing in toddlers is affected by sub-segmental phonological detail.

In this set of simulations, we examine the adaptations of the TRACE architecture that are needed to simulate the White and Morgan (2008) results, and explore the ramifications of these adaptations for interpreting their experimental findings. At first blush, modelling White and Morgan’s (2008) results in TRACE would seem to be a trivial matter: TRACE exploits sub-segmental features to define the phonological form of a word. Activation of a lexical entry in TRACE can be expected to reflect the severity of the perturbation of these sub-segmental features, and hence the amount of target looking for a mispronounced word (Dahan et al., 2001b). We will see, however, that asymmetries in the cohort competition effects discovered in the previous set of simulations conspire against a straightforward interpretation of White and Morgan’s (2008) results, and lead to a re-evaluation of the dynamics of lexical processing in the early infant lexicon.

### Method

We used the stimuli described in Experiment 1 of White and Morgan (2008), reproduced in Table 5, with the exception of the word “cookie”, which is not present in the British version of the CDI that we used to create the new TRACE lexicons. Since the distractor is name-unknown in the White and Morgan (2008) experiment, the activation level associated with the novel object on display is set to zero. Note, however, that due to the application of Luce’s rule, both images share some amount of the total looking time spent during each trial. Simulations were run with the 18-month-lexicon to mimic the behaviour of 19-month olds.

**Table 5:** Correctly pronounced and mispronounced labels presented to infants in Experiment 1 of White and Morgan (2008). Note that only the onset consonant is changed and that the medial vowel remains unchanged. The unfamiliar words used by White and Morgan (2008) are not considered because they do not compete for recognition in TRACE. The table also includes the cohort size and Mean Neighbourhood Densities as a function of pronunciation type for the stimuli used in White and Morgan (2008).

Correct pronunciations	Mispronunciations		
	1-Feature	2-Feature	3-Feature
keys/kiːz/	teys	deys	zeys
book/b*℧*k/	dook	took	sook
bear/b*ε*ː/	gear	tear	sear
foot/f*℧*t/	soot	zoot	goot
car/kɑ/	par	dar	zar
ball/bɔːl/	gall	kall	sall
bird/bəːd/	gird	kird	sird
bottle/bɒtl/	gottle	kottle	sottle
shoe/ʃuː/	foe	voe	goe
cup/kʌp/	tup	bup	vup
hand/hand/	fand	zand	dand

*Mean cohort size (SD)*			
18.7 (12.1)	7.7 (7.2)	11.7 (9.9)	4.4 (2.5)

*Mean Neighbourhood Densities (MND)*			
0.27 (0.65)	1.45 (0.52)	1.45 (0.69)	1.27 (0.47)

### Results

**Fig. 9:**
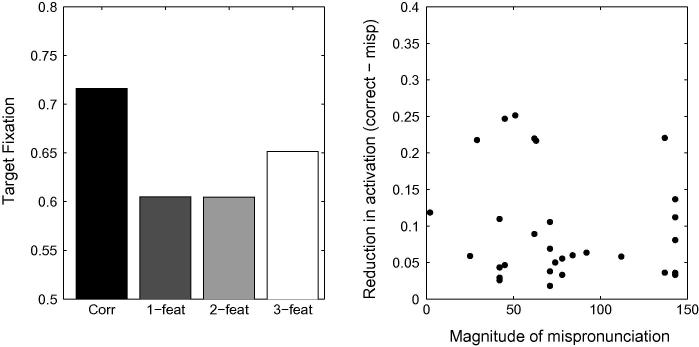
Left Panel: Simulation of White and Morgan (2008) with TRACE’s default parameters. The unbalanced cohort sizes in each condition interferes with the bottom-up activation flow that otherwise favours graded sensitivity to the severity of the mispronunciations. In particular, looking times in the three-feature mispronunciation condition are longer than in the one- and two-feature mispronunciation conditions. Right panel: Mispronunciation effect (reduction in activation due to the mispronunciation) as a function of the magnitude of the mispronunciation in TRACE’s feature space. No correlation is observed between looking times and the severity of mispronunciations.

First, we ran simulations with TRACE’s default parameters for the same stimuli used by White and Morgan (2008). The top panel of Fig. 9 depicts the proportion of looking time associated with the target in the correct, 1-feature-, 2-feature- and 3-feature-mispronunciations. No graded sensitivity is observed as a function of the severity of mispronunciation. Since the auditory metrics used by White and Morgan (2008) to derive the severity of mispronunciation may differ slightly from TRACE’s, we also evaluate the impact of the severity of mispronunciations on the level of activation of the target words within TRACE’s metrics. The right hand panel of Fig. 9 depicts the reduction in activation level as a function of the magnitude of the mispronunciation (Euclidean distance between the two phonemes in TRACE’s feature space) for all stimuli. The absence of any correlation indicates that activation levels of target words in TRACE are not directly sensitive to the severity of mispronunciations, in contrast to White and Morgan’s (2008) findings.

Closer examination of the stimuli used by White and Morgan (2008) reveals that the number of cohort competitors in the typical lexicon of an 18-month old differs dramatically with mispronunciation type. Table 5 presents an analysis of the cohort size associated with correct pronunciations and each mispronunciation type. It is apparent that 3-feature mispronunciations have far fewer cohort competitors than any of the other mispronunciation conditions. An item-based analysis-of-variance of the number of cohort competitors across types of pronunciation yielded a main effect of pronunciation condition (*F* = 5.53, *df* = 3, *p* = .0028). Two feature mispronunciations have marginally more cohort competitors than 1-feature mispronunciations (*t* = 1.34, *df* = 10, *p* = .21, n.s.), and more importantly, more than 3-feature mispronunciations (*t* = 2.40, *df* = 10, *p* = .038).^8^

An important characteristic of TRACE is that it implements competition within the different layers of the network. As a consequence, cohort competitors impact the activation levels associated with a target word. A low number of cohort competitors leads to reduced inhibition which, in turn, leads to higher activation of the target word. Experimental evidence that competition is not tied to visible distractors is provided by Magnuson, Dixon, Tanenhaus, and Aslin (2007), who showed that cohort and neighbourhood density influence fixation behaviour even when there are no phonologically related distractors in the display. For the stimuli used by White and Morgan (2008), we expect the cohort competition in TRACE to interfere with any mispronunciation effect. In particular, the low number of cohort competitors in the case of the 3-feature mispronunciation would lead to an *increase* in the activation of the target word, rather than to a *decrease* in its activation level. Clearly, this outcome would be incommensurate with White and Morgan’s (2008) finding of a graded sensitivity to severity in the mispronunciation and explains why a graded sensitivity to the severity of mispronunciations was not observed with TRACE’s default parameters. Therefore, we conducted further simulations to identify conditions under which TRACE does display graded sensitivity to the severity of mispronunciations, given the stimuli used by White and Morgan (2008).

### The role of inhibition

TRACE possesses many parameters that influence the dynamics of its speech perception characteristics. All of them impact the trajectories of word activation levels (see McClelland and Elman, 1986, McMurray et al., 2010). An exhaustive search in this multi-dimensional parameter space is beyond the scope of the present work. However, the manner in which the previous simulation failed to produce the desired effect suggests that *cohort competition* is at the heart of the problem. We investigate four factors that can be expected to reduce the impact of this competition.

1. The strength of feedforward connections in the network.
2. The cohort balance in TRACE’s lexicon.
3. Lateral inhibition between lexical representations.
4. Lateral inhibition between phonological representations.

#### Reduced phoneme-to-word connection strengths

**Fig. 10:**
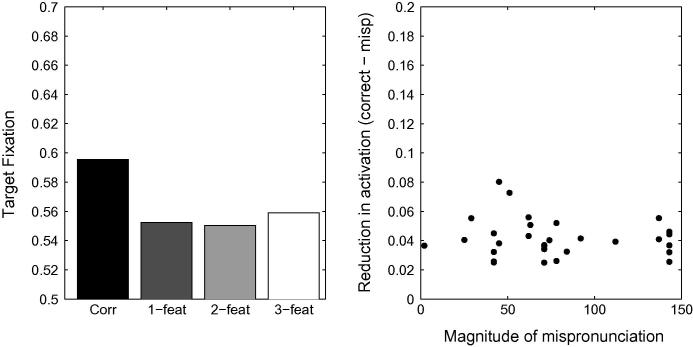
Left Panel. Simulation of White and Morgan (2008) using TRACE with reduced phoneme-to-word connection strengths. Graded sensitivity to mispronunciation is absent. Right Panel. Reduction of looking times does not correlate with mispronunciation severity.

A first candidate for modification is to reduce the strength of the connections between phonemes and words, thereby implementing the view that word-form representations do not possess adult-like strengths during the early stages of lexical development. A reduction in phoneme-to-word connection strength will prevent the network from committing too early, and sometimes irreversibly, to an individual lexical item based on the presentation of the first phoneme only. Individual lexical items will only become predominantly active after several phonemes have been processed. Phoneme-to-word connection strengths were reduced to 0.01, five times weaker than their default values of 0.05. Fig. 10 depicts the mean activation values associated with correct pronunciations, 1-feature, 2-feature and 3-feature mispronunciations. It is apparent that graded sensitivity is not achieved through this modification to TRACE: 3-feature mispronunciations still attract longer looking times than 2-feature mispronunciations. A comparison of the reduction of activation levels and the magnitude of the mispronunciations in TRACE’s feature space reveals no correlation (R=-0.12,p=0.53).

The imbalance in cohort sizes still results in a non-graded sensitivity to the severity of mispronunciations. A reduction in phoneme-to-word connection strength is, on its own, insufficient to capture the experimental data.

#### Balancing the lexicon

The results obtained by White and Morgan (2008) seem entirely intuitive; graded mispronunciations lead to a gradation in looking times towards the target. The core of the difficulty in capturing these experimental results by simulation is the imbalance in cohort sizes in the lexicon used in TRACE, associated with the stimuli used in the experiment. TRACE’s lexicons are obtained by compiling CDI reports, thus permitting the construction of a standard vocabulary of words known by most infants at 18 months of age. However, CDI reports only provide a lower bound to the number of words known at any given age (Mayor & Plunkett, 2011). One might argue that real lexicons are more evenly balanced with respect to cohort sizes and that the stimuli used by White and Morgan (2008), viewed from the perspective of balanced lexicons, give rise to similar levels of competition between words across experimental conditions which, in turn, would allow the network to display graded sensitivity.

**Fig. 11:**
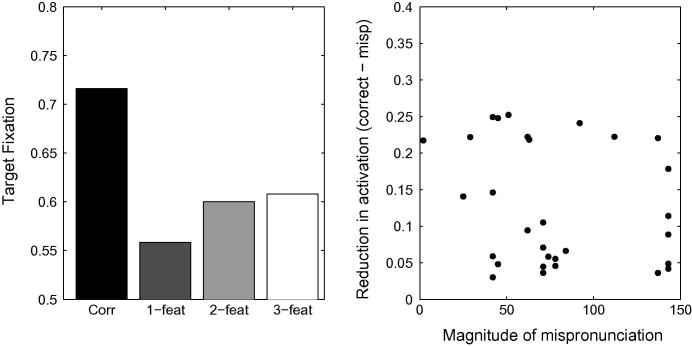
Left Panel. Simulation of White and Morgan (2008) using TRACE with a more balanced lexicon. The network fails to display graded sensitivity to mispronunciations. Right Panel. Correlation between a reduction in target looking and mispronunciation severity is not significant.

In order to test this hypothesis, we added words to TRACE’s 18-month lexicon so that v-, g-, and s-onsets, over-represented in the 3-feature mispronunciation condition, would belong to cohorts of similar sizes as the other onsets. *Sun, sister, sweets, sky, snow, sleep, squirrel, stop, soap, stove, star, spider, good, go, gloves, garage, goose, game, grass, girl, glasses, vegetable, van, vanilla, vase* and *very* were added to the lexicon to this end, with an intermediate frequency level of 300. Cohort sizes across the different mispronunciation conditions were similar (respectively 12.1, 12.2 and 11.9), and did not differ significantly (an ANOVA of the cohort sizes revealed no effect of pronunciation condition; *F* = 1.51, *df* = 3, *p* = .23). Furthermore, cohort sizes associated with 2-feature and 3-feature conditions did not differ significantly (*t* = 0.088, *df* = 10, *p* = .93, n.s.). Fig. 11 displays looking times at the target, when simulated with the new lexicon, while all other parameters are kept constant. Again, graded sensitivity does not emerge, despite the fact that cohort sizes are equivalent across experimental conditions.

This finding seems to undermine the claim that the imbalance in cohort sizes prevents TRACE from capturing the graded sensitivity to mispronunciation severity observed by White and Morgan (2008). However, closer examination reveals that the overall density of the phonological neighbourhood, not just the cohort size of potential word forms impacts lexical activation. For example, words whose mispronunciations have an s-onset, lead to higher levels of activation than when the onset is k-, despite having equivalent cohort sizes. Thus, a 2-feature mispronunciation of *bottle*, e.g., *kottle*, leads to a very reduced level of activation for bottle, whereas *sottle*, a mispronunciation of higher magnitude, activates *bottle* to a higher degree. Rather than a single onset phoneme, it is the onset density (the first few phonemes) that drive this effect. In this example, s-onset words sparsely populate the space around *sottle*, whereas multiple k-onset words are very close to *kottle* in TRACE’s phonemic/featural space (e.g., *cot, cat, car, cup, cow, kitchen, coat,* and many more). Regions in lexical space have uneven densities which, in turn, lead to uneven levels of competition for different test words. Unless infant vocabularies are very well balanced, not only at the level of onsets,^9^ but also in terms of lexical densities extending to the general phonological neighbourhood, TRACE is unlikely to produce such a clear graded sensitivity to the severity of mispronunciation as reported by White and Morgan (2008).

#### Reduced lexical inhibition

Next, we investigate the impact of reducing the level of lexical inhibition. Both theoretical and experimental considerations motivate this adaptation of TRACE: Lexical inhibition may be reduced in infancy due to the sparseness of the lexical space (see Gaskell & Marslen-Wilson, 1997 for related discussion regarding adult word recognition). Several recent experimental findings provide evidence that word-to-word interactions do not reach adult levels of competition before about 21 months of age. For example, Arias-Trejo and Plunkett, 2009, Styles and Plunkett, 2009 used a semantic priming task with infants to demonstrate evidence for lexico-semantic networks in 21- and 24-month old infants. However, they failed to find evidence of semantic priming in 18-month olds. Arias-Trejo and Plunkett (2009) suggest that entries in the 18-month old lexicon may be best characterised in terms of *lexical islands* that are not in competition with each other because they are unconnected. More direct evidence is provided in a phonological priming task (Mani & Plunkett, 2011) conducted with 18- and 24-month old infants. Mani and Plunkett (2011) reported cohort competition effects in 24-month olds (less target looking for words from large cohorts than words from small cohorts) but no cohort competition effects for 18-month olds, pointing to an absence of lexical competition at the younger age. These age differences in cohort competition effects may be driven by differences in the vocabulary sizes of the infants involved in the study, even though both age groups were tested on the same set of words, and only words familiar to the infants were included in the analyses. Note that Table 3 indicates a substantial increase in cohort size, and hence potential competition effects, between 18- and 24-months of age. This set of findings, together with the findings from Arias-Trejo and Plunkett, 2009, Styles and Plunkett, 2009, provide a convergent rationale for reducing lexical competition in the simulation of White and Morgan’s 19-month old infants.

**Fig. 12:**
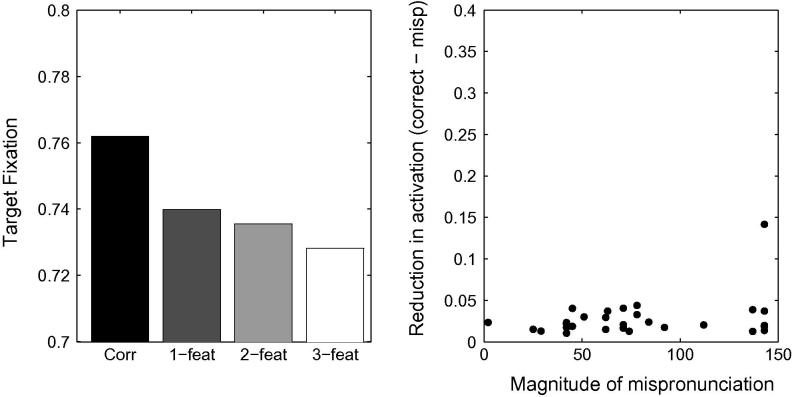
Left Panel. Simulation of White and Morgan (2008) using TRACE with reduced lexicon competition. Cohort competition effects are reduced and the bottom-up activation flow favouring graded sensitivity to the severity of the mispronunciations is not disrupted. Right panel. Mispronunciation effect (reduction in activation due to the mispronunciation) as a function of the magnitude of the mispronunciation in TRACE’s feature space. A weak, non-significant, correlation is observed between looking times and the severity of mispronunciations.

The left hand panel of Fig. 12 displays the proportion of target looking in TRACE associated with the stimuli used by White and Morgan (2008) for correct, 1-feature, 2-feature and 3-feature mispronunciations when lexical inhibition is essentially turned off (*C* = 0.0001).^10^ A graded sensitivity to the severity of mispronunciations emerges, similar to that observed for the 19-month olds tested by White and Morgan (2008). However, correlations between the reduction of activation levels associated with target words and the magnitude of the mispronunciations in TRACE’s feature space did not reach significance (*p* = .13, see right hand panel of Fig. 12). For C⩾0.001, cohort competition effects counteract the effect of mispronunciation such than the activity level associated with the 3-feature mispronunciations is higher than the activity level associated with the 2-feature mispronunciations.

**Fig. 13:**
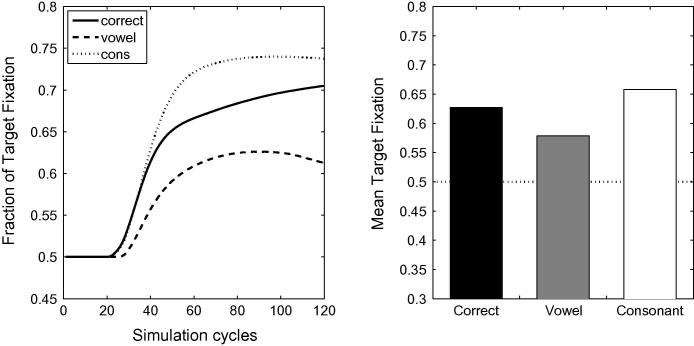
Simulation 1 of Mani and Plunkett (2007) for 18-month lexicons, with weak lexical inhibition. Reduced lexical inhibition maintains word recognition for correct pronunciations and both vowel and consonant mispronunciations. Note, however, that looking time is more robust for consonant mispronunciations than for correct pronunciations (see text for a detailed explanation).

A substantial reduction of lexical inhibition leads to increased levels of activation for all words in the lexicon, due to the absence of regulatory mechanisms other than lexical decay. This loss of tuning makes word recognition more difficult owing to the multiplication of potential candidates. In order to evaluate the capacity of the network to recognise words under these conditions, we re-ran Simulation 1 of Mani and Plunkett (2007) with reduced lexical competition. Fig. 13 displays time course plots and mean activation levels associated with correct pronunciations as well as both onset consonant and medial vowel mispronunciations, for the 18-month lexicon model. In all cases, target looking times exceed chance levels, suggesting that reduced lexical inhibition does not disrupt the capacity of the model to recognise words. However, consonant mispronunciations lead to more robust target preferences than correct pronunciations — a counter-intuitive and unsatisfactory result. The effect can easily be understood, once it is recalled that in this experiment the labels for the target and distracter begin with the same onset consonant: in onset consonant mispronunciations, the initial phoneme does not activate the target nor the distractor. However, for the remaining part of the word, the target overlaps with the input whereas the distractor has no overlap, resulting in robust target looking. In contrast, correct pronunciations activate both the target and the distractor from the first phoneme. In the absence of strong lexical inhibition, both words maintain a high level of activation, resulting in a higher level of competition when applying the Luce choice rule and in less robust target preferences. Enhanced target looking for onset mispronunciations compared to correct pronunciations is clearly incompatible with the experimental findings.^11^ However, as lexical inhibition is progressively increased, correct pronunciations exceed looking times associated with consonant mispronunciations. Any attempt to accommodate White and Morgan’s findings by suppressing lexical inhibition in TRACE, and by implication lexical competition in 19-month-olds, requires the identification of an intermediate level of inhibition compatible with experimental results from both Mani and Plunkett, 2007, White and Morgan, 2008.

#### Reduced phoneme inhibition

**Fig. 14:**
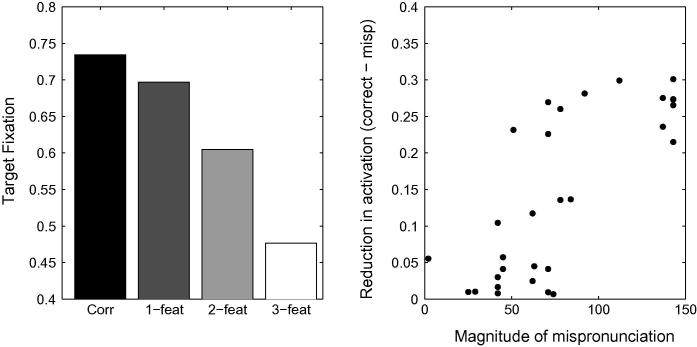
Left Panel: Simulation of White and Morgan’s (2008) findings in TRACE with no phoneme inhibition. Cohort competition effects are reduced and bottom-up flow of activation favouring graded sensitivity to the severity of the mispronunciations is well-established. Right panel: Mispronunciation effect (reduction in activation due to the mispronunciation) as a function of the magnitude of the mispronunciation in TRACE’s feature space. A strong, significant, correlation is observed between target preference and the severity of mispronunciations.

Another manipulation that can alter the influence of imbalanced cohort sizes when simulating White and Morgan’s (2008) findings is to reduce phoneme inhibition. McMurray et al. (2009) argued that phoneme-level inhibition in TRACE is incompatible with recovery from “lexical garden-paths” initiated by ambiguous phonemes early in a word. We consider next the impact that a complete absence of phoneme-level inhibition has on simulations of White and Morgan’s (2008) findings. The left hand panel of Fig. 14 depicts the proportion of looking time at the target when correctly pronounced, and with three levels of mispronunciation severity, when phoneme level inhibition is eliminated in TRACE. A clearcut, graded reduction in activation level emerges as the number of feature changes increases. Furthermore, the right hand panel of Fig. 14 indicates that, within TRACE’s feature metrics, a significant correlation $\left(R=0.753\text{,}p=1.56\times 10^{-6}\right)$ is present between the magnitude of the mispronunciation and its impact on activation levels. Cohort competition effects are effectively eliminated and the bottom-up flow from the feature level to the lexical level, via the phoneme level, is undisrupted by cohort members.

**Fig. 15:**
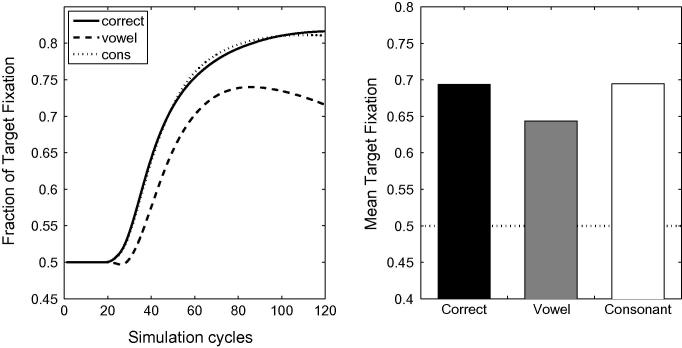
Simulation of Experiment 1 of Mani and Plunkett (2007) for 18-month lexicons, with no phoneme inhibition. Absence of phoneme inhibition maintains target recognition for correct pronunciations and both vowel and consonant mispronunciations. Target preference is slightly enhanced for consonant mispronunciations over correct pronunciations.

Again, it is necessary to check that removing phoneme-level inhibition in TRACE does not disrupt the capacity of the network to recognise words. We re-ran the simulation of Mani and Plunkett (2007) for the 18-month lexicon model but with phoneme-level inhibition eliminated. Fig. 15 shows significant target looking in all pronunciation conditions (correct and both consonant and vowel mispronunciations). Similar to the effect of reducing lexical-level inhibition, consonant mispronunciations possess a slight advantage over correct pronunciations which activate both target and distracter. The absence of phoneme competition allows for a wider activation of phonemes, in turn activating a larger set of words in TRACE’s lexicon. The enhanced level of activation associated with the distracter leads to reduced target looking for correct pronunciations, via the Luce choice rule.

#### Complex interplay of parameters

Of the four parameter manipulations investigated, reduction of lexical or phoneme inhibition both succeeded in simulating graded sensitivity to the severity of target mispronunciations, as required by White and Morgan’s (2008) findings. However, success was achieved at a cost: TRACE no longer had the capacity to distinguish appropriately between correct and onset mispronunciations, as required by Mani and Plunkett’s (2007) findings and many others. These findings seem to require distinct characteristics from the model. We attempted, therefore, to identify a middle ground where intermediate levels of inhibition might allow TRACE to capture both experimental findings. A limited search in the space of parameters concerning phoneme and lexical inhibition, however, failed to identify a single set of parameters that could simultaneously account for the findings of Mani and Plunkett, 2007, White and Morgan, 2008. Table 6 reports the explored set of parameter values and their overall agreement with experimental data. In essence, if either phoneme or lexical inhibition remains weak, a graded sensitivity to the severity of mispronunciations will emerge. However, mimicking Mani and Plunkett (2007) requires higher levels of phoneme *and* lexical inhibition in order to maintain longer looking times for the correct pronunciation over the onset mispronunciations.

**Table 6:** Inhibition space.

Phoneme inhibition	Lexical inhibition	Agreement with Mani and Plunkett (2007)	Agreement with White and Morgan (2008)
0	0.03	No	Yes
0.004	0.02	No	No
0.004	0.03	Not quite	Not quite
0.008	0.03	Yes	No
0.01	0.02	Yes	No
0.02	0.02	Yes	No
0.04	0.001	No	Yes
0.04	0.003	No	Yes
0.04	0.03	Yes	No

**Fig. 16:**
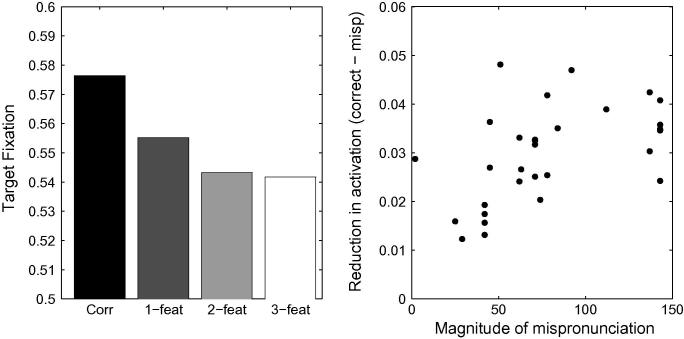
Left Panel: Simulation of White and Morgan’s (2008) findings in TRACE where phoneme-to-word connection strengths, phoneme and lexical inhibition are all reduced. Right panel: Mispronunciation effect (reduction in activation due to the mispronunciation) as a function of the magnitude of the mispronunciation in TRACE’s feature space. A strong significant correlation is observed between target preference and the severity of mispronunciations.

**Fig. 17:**
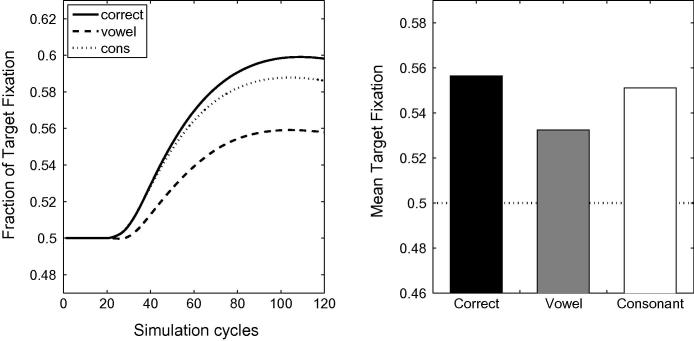
Simulation of Experiment 1 of Mani and Plunkett (2007) for the 18-month lexicon, where phoneme-to-word connection strengths, phoneme and lexical inhibition are all reduced. Onset consonant mispronunciations attract less looking times at the target than correct pronunciations, as described in Mani and Plunkett (2007).

A solution to this conundrum can be found by lowering the phoneme-to-word connection strengths while maintaining low levels of inhibition. In so doing, the weak phoneme-to-word connections prevent the system from committing too early based on the initial phoneme, consequently reducing cohort competition effects that would otherwise undermine graded sensitivity to mispronunciations. Inhibition parameters do not then need to be reduced so dramatically in order to capture White and Morgan’s (2008) findings. Moderate levels of inhibition at the phoneme and lexical level ensure that consonant mispronunciations attract less looking times than correct pronunciations. TRACE’s performance with such a combination of parameters is depicted in Fig. 16. Phoneme inhibition is set at 0.004, lexical inhibition to 0.02 and phoneme-to-word weights to 0.01. A well-formed, graded sensitivity to the severity of mispronunciations is obtained and an analysis of the reduction of looking times as a function of the magnitude of mispronunciation (lower panel of Fig. 16) shows the effect is robust (*r* = .497, *p* = .0052). The same combination of parameters enables the network to capture Mani and Plunkett’s (2007) findings, in having reduced looking times for mispronounced words as compared with correct pronunciations, as shown in Fig. 17.

### Discussion

White and Morgan (2008) reported a graded sensitivity in 19-month old infants to the severity of the mispronunciation of a target word and argued that this finding demonstrated fine-grained sensitivity at the sub-segmental level. The gradual decrease in looking time at the target object as the number of modified features increased was observed despite the fact that the number of cohort competitors for mispronunciations, as evaluated by an analysis of CDI reports, was smaller for the 3-feature mispronunciations than for the 2-feature mispronunciations. Competition between word activation levels in TRACE has an opposite effect on target word activation for the stimuli used by White and Morgan (2008), leading to an apparent incompatibility between White and Morgan’s findings and the predictions of TRACE. We therefore investigated a variety of factors that might circumvent the misalignment between the experimental and computational results.

Taken individually, neither a reduction in phoneme-to-word connection strengths, in lexical-level inhibition, the removal of phoneme-level inhibition, nor a finer-grained estimate of vocabulary composition in infancy could fully account for the graded sensitivity to mispronunciations described in White and Morgan (2008) while also capturing the findings that both onset consonant and medial-vowel mispronunciations lead to a reduction in target preferences reported by Mani and Plunkett (2007). A satisfactory account of both sets of results appears to require manipulation of *combinations* of parameters within TRACE.

One such set of parameters, in which we reduced phoneme-to-word connection strengths *and* inhibition at the phoneme and at the lexical level was able to capture both sets of experimental findings. Lower levels of inhibition prevented the uneven lexical densities from disrupting a monotonic degradation in looking time with increasing mispronunciation severity, whereas weaker phoneme-to-word connections prevented the network from committing too early upon the presentation of the initial phoneme.

Manipulation of these parameters in TRACE constitute a plausible exploration of factors that might impact infant word recognition: First, connections between the constituent segments of lexical items are likely to strengthen with experience permitting faster and more robust word recognition (Fernald, Pinto, Swingley, Weinberg, & McRoberts, 1998). Second, recent evidence suggests that inhibitory lexical processes emerge during the latter half of the second year (Arias-Trejo and Plunkett, 2009, Arias-Trejo and Plunkett, 2013, Mani and Plunkett, 2011). These studies report that lexical competition effects are apparent by 24-months of age, but not at 18-months of age. If the inhibition hypothesis holds, we would predict, therefore, that when a task like White and Morgan’s (2008) study is conducted with 24-month-old infants, then the impact of severity of mispronunciation is likely to diminish.

### Testing the inhibition hypothesis

Bailey and Plunkett (2002) also manipulated mispronunciation severity of word onset consonants (1-feature or 2-feature) in an IPL study with 18- and 24-month-old infants. Unlike the White and Morgan (2008) study, the distracter image was a picture of a name-known object and hence had the potential to function as a lexical competitor. In contrast to the Mani and Plunkett (2007) studies, the labels for the distracter images in Bailey and Plunkett (2002) did not overlap phonologically with the target labels. When the TRACE model of word recognition is supplemented with the Luce choice rule, absence of phonological overlap renders lexical competition from the distracter label ineffective, since TRACE evaluates competition on the basis of phonological information alone. From the perspective of TRACE, the Bailey and Plunkett (2002) study is quite similar to White and Morgan (2008) in that the target is the only potential match for TRACE. We have suggested that lexical competition is absent in the lexicons of 18-month olds but manifest for 24-month olds. Consequently, we would expect the 18-month olds in Bailey and Plunkett (2002) to behave similarly to the 19-month olds in the White and Morgan (2008) study but, potentially, lexical and phonemic competition to interfere with the impact of mispronunciation severity for the 24-month olds.

**Fig. 18:**
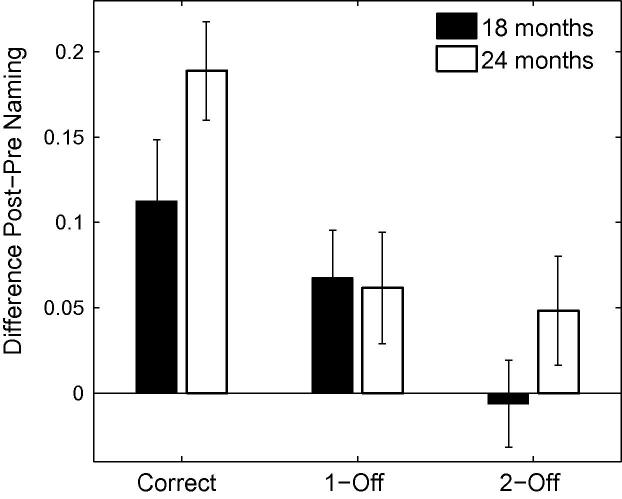
Target preferences for correct, 1-feature and 2-feature mispronunciations of well-known words used by Bailey and Plunkett (2002).

In their study, Bailey and Plunkett (2002) considered names of objects well-known to the infants as well as names only recently acquired, as assessed by a longitudinal CDI survey for individual infants. A re-analysis of the looking behaviour in response to targets that were *well-known* to the infants yielded a pattern of results similar to White and Morgan (2008) for the 18-month olds but a different pattern for the 24-month olds. This re-analysis of the target preferences of the infants in the Bailey and Plunkett (2002) study is depicted in Fig. 18. An ANOVA comparing the differences in target preferences between pre-naming and post-naming trial phases revealed a main effect of pronunciation type (correct, 1-feature and 2-feature) for both the 18-month olds (*F* = 3.96, *df* = 2, *p* = .024) and the 24-month olds (*F* = 6.15, *df* = 2, *p* = .004). A linear regression of target looking against pronunciation type also yielded a significant negative correlation for both the 18-month old infants (Correlation coefficient = -0.318, CI = [−0.512 −0.093], *p* = .007) and the 24-month olds (Correlation coefficient = −0.352, CI = [−0.540 −0.131], *p* = .0024). Planned comparisons between pronunciation conditions for the 18-month olds revealed no significant difference between correct and 1-Off mispronunciations (*t*(23) = 1.03, *p* = .32), a marginally significant difference between 1-Off and 2-Off mispronunciations (*t*(23) = 1.84, *p* = .078), and a significant difference between correct and 2-Off mispronunciations (*t*(23) = 2.62, *p* = .015). In contrast, for the 24-month olds, the mispronunciation effect is driven entirely by large differences in target preferences between correct pronunciations and either level of mispronunciation (Correct-1Off: *t*(23) = 3.56, *p* = .0017; Correct-2Off: *t*(23) = 3.33, *p* = .0029). No systematic difference in target preference is observed between 1-Off and 2-Off mispronunciations (*t*(23) = 0.297, *p* = .77). This would suggest that the strong and regular reduction in target preferences as mispronunciations become increasingly severe at 18 months is less marked at 24 months, when lexical inhibition is likely to be stronger (Arias-Trejo and Plunkett, 2009, Arias-Trejo and Plunkett, 2013, Mani and Plunkett, 2011).

Table 7 shows that, for the 18-month olds, 1-feature mispronunciations have slightly more cohort competitors than 2-feature mispronunciations which, in the face of lexical competition, should result in diminished target preference for the former compared to the latter. However, the 18-month olds in Bailey and Plunkett’s study exhibit graded sensitivity to severity of mispronunciation, consistent with the White and Morgan’s findings, and again pointing to an absence of lexical competition consistent with other studies (Arias-Trejo and Plunkett, 2009, Mani and Plunkett, 2011). In contrast, the 24-month olds did not exhibit such a regular, graded sensitivity, owing potentially to complex interference effect with other words in the lexicon at 24 months of age, driven by the presence of lexical competition.

**Table 7:** Cohort size as a function of pronunciation type for the stimuli used in Bailey and Plunkett (2002).

	Correct pronunciations	Mispronunciations	
		1-Feature	2-Feature
18 Months	16.66	8.71	6.35
24 Months	22.17	10.60	10.94

In summary, this re-analysis of the Bailey and Plunkett (2002) study provides further support for White and Morgan’s (2008) findings when viewed from the phonological word recognition framework offered by TRACE, and is consistent with the claim that lexical competition emerges some time during the second half of the second year of life, such that competition from cohort and other phonological neighbours can impact and interfere with graded sensitivity to mispronunciation severity of familiar words. Of course, this does not rule out the possibility that other contributing factors, such as phoneme-level inhibition, reinforcement of word forms, or a more realistic account of lexical composition in infancy, also play a prominent role. However, we suggest that a demonstration of graded sensitivity to mispronunciation severity does not necessarily indicate that “learners [are] unwilling to accept mispronunciations as labels of known objects” (White & Morgan, 2008, p. 128), but rather may reflect a *goodness-of-fit* evaluation of the heard label to the name of the familiar target object, as implemented in this TRACE simulation. In fairness to White and Morgan (2008), we should also point out that our simulations do not challenge one of the main claims of their work that “the architecture underlying lexical representation and processing is adult-like by 19 months” (White & Morgan, 2008, p. 128), though we would add that this architecture does not yet appear to incorporate lexical competition.

Additional motivation for varying the lexical competition parameter in TRACE comes from the neuropsychological literature. For example, Mirman, Yee, Blumstein, and Magnuson (2011) have shown that slower deactivation of lexical competitors could account for increased cohort competition in Wernicke’s aphasics. It is noteworthy that deviations from normal patterns of rhyme priming^12^ are apparent in fluent and non-fluent aphasics. Milberg, Blumstein, and Dworetzky (1988) showed the fluent and non-fluent aphasics fail to demonstrate graded sensitivity in a rhyme priming task. However, they fail in different ways. Fluent aphasics seem to accept any rhyme as a viable token of the prime word (*wat* or *gat* for *cat*) whereas non-fluent aphasics appear to require a precise rendition of the prime word for any priming to occur. In discussing a potential application of TRACE to their findings, the authors argue that:

“the impairments displayed by the aphasic patients may reflect impairments in the processing mechanisms contributing to lexical access. (…) The fluent aphasics could be characterized as having a decreased threshold of sensitivity for lexical access. Thus, they would show a lessened sensitivity to phonological distortion, subsequently accessing more words in the lexicon than normal. In contrast, the nonfluent aphasics could be characterized as having an increased threshold of sensitivity to lexical access. Thus, they would show an increased sensitivity to phonological distortion, subsequently accessing fewer words in the lexicon than normal.” Milberg et al. (1988, pp. 291–292).

From the perspective of TRACE, thresholds of sensitivity are a metric of lexical competition effects. Fluent aphasics seem to lack lexical competition whereas non-fluent aphasics are apt to over-apply it. It is a laudatory aspect of TRACE that it can be expanded to encompass aspects of both developmental and neuropsychological aspects of word recognition, despite its original implementation as a non-developmental model of normal adult word recognition. We now turn to the implications that the TRACE model of word recognition has for aspects of word learning. Our focus is upon early word learning though we assume that these implications will extend to adult word learning too.

## Word Learning and lexical competition

Swingley and Aslin (2007) investigated the impact of phonological neighbourhoods on early word learning. Seventeen- to twenty-month-olds were taught novel labels for novel objects. The novel labels were either similar to a familiar word (referred to as a novel neighbour) or dissimilar (non-neighbour). After infants were taught a novel neighbour (a rhyme) and a novel non-neighbour, they were tested for comprehension using an intermodal preferential looking task. In a first block of testing, they were presented with the two novel objects while hearing one of the novel words. Infants showed increased looking times to the target (the appropriate novel object) only when the novel word was a non-neighbour. In a second block, infants were presented with a familiar object and the object whose novel label was either a neighbour of the name of the familiar object (*tog-dog* or *gall-ball*) or a non-neighbour (*meb-car* or *shang-baby*), together with the appropriate previously taught novel label. Infants showed increased looking times to the novel object in both cases, although the magnitude of the naming effect was greater for the non-neighbour. Swingley and Aslin (2007) concluded that novel words that are neighbours of familiar words are more difficult to learn than non-neighbours.

TRACE is not a model of word learning. However, we can use TRACE to compute the likely levels of activation of familiar words when presented with a novel word. The linking hypothesis is that high levels of activation of familiar word candidates in TRACE, on presentation of a novel word, hinders learning of the novel word, because activation of a familiar word renders it a plausible label in the current learning situation. Thus, TRACE allows us to evaluate, from a computational perspective, Swingley and Aslin’s (2007) claim that “that word learning in young children, as in adults, relies not only on the discrimination and identification of phonetic categories, but also on evaluating the likelihood that an utterance conveys a new word” (ibid. p. 99). Because TRACE is not a model of word learning, our simulations explore the alternative possibility that Swingley and Aslin’s (2007) findings can be explained in terms of mispronunciation effects rather than a differential capacity to learn novel non-neighbour as compared to neighbour words. In the light of previous simulations where it was necessary to reduce lexical competition to simulate White and Morgan’s (2008) findings for 19-month olds, we explore two configurations of TRACE, first with default levels of competition and then with reduced competition, as reported earlier in Fig. 16, Fig. 17.

### Method

We used the stimuli described in Swingley and Aslin (2007); *tog* and *gall* for neighbours and *meb* and *shang* for non-neighbours. The simulations used the 18-month lexicon described earlier (see Table B.9). In a first simulation, we assume that no learning has occurred and that the activation level associated with the novel object on display is set to zero. In a second simulation, we continue to assume that no word learning has occurred but that the novel object attracts additional looking, owing to a novelty-based salience. In this simulation, we assume that the novel object has a non-linguistic-based activation level (set to 0.2) used in Luce’s rule when computing relative looking time to the novel and to the familiar object. In a third simulation, we assume that *both* the novels words, neighbour and non-neighbour, have been learnt in the first block of Swingley and Aslin’s experiment. This is achieved by introducing the novels words as low-frequency words into the TRACE lexicon.^13^ In this third simulation, both images on display possess an entry in TRACE’s lexicon. Together, these three simulations allow for a comparison of TRACE’s capacity to model the experimental results when learning is not implemented and after learning has occurred. All three simulations attempt to provide a different interpretation to that of Swingley and Aslin (2007) where it is claimed that only the novel, non-neighbour word is learnt.

In all simulations, we assume that total looking time is constant and split between the name-known and the novel pictures. To enable direct comparison with Swingley and Aslin’s results, where a salience-corrected measure is reported, we added 0.5 to Swingley and Aslin’s measure, thereby assuming that looking time in the pre-naming phase was split evenly between the two objects.

### Results

#### With default parameters

**Fig. 19:**
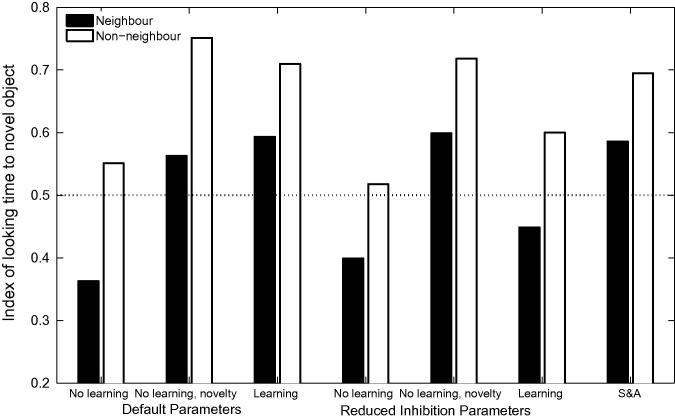
Simulations of Swingley and Aslin (2007) where (i) novel words are not part of the lexicon (no learning has occurred) and where there is no intrinsic salience associated with either object on display. Presentation of novel words that are neighbours of the familiar picture’s label (weak mispronunciation) activate that label more than when a non-neighbour word is presented (strong mispronunciation), leading to a decrease in looking time to the novel object. (ii) A novelty-based salience leads to longer looking times towards the novel object when named, providing a qualitative match to Swingley and Aslin’s results. (iii) When novel words are introduced into the lexicon as low-frequency entries, both images can be associated with a lexical item and the Luce choice rule can be applied. Target looking patterns closely match the experimental results.

In the first simulation, no word learning has occurred and it is assumed that no intrinsic salience of the objects supplements the activation level of the lexical entries. In the neighbour condition (see Fig. 19), the distractor (i.e., the familiar object) attracts substantial looking time since the label uttered (e.g., tog) is only a small mispronunciation of the label associated with the distractor image (e.g., dog). Owing to the lack of activation associated with the novel item, presentation of neighbour words leads to a decrease in looking time to the novel object. In the non-neighbour condition, the activation level associated with the familiar object (e.g., baby) is very low due to the absence of phonological overlap to the label uttered (e.g., shang), thus resulting in higher looking times towards the novel object than in the neighbour condition, matching qualitatively, but not quantitatively, Swingley and Aslin’s results.

In a second simulation (see Fig. 19), it is assumed that a novelty-based salience induces a non-linguistic activation level associated with the novel object. As in the previous simulation, looking times towards the novel object are longer in the non-neighbour trials than in the neighbour trials. Note that the saliency adjustment shifts all looking times towards a more novelty-based looking behaviour, but does not qualitatively change the pattern of looking preferences. Reduced looking time in the neighbour condition is driven by a ‘mispronunciation effect’ of the neighbouring word, thereby driving attention away from the novel object.

Finally, Fig. 19 depicts activation values when *both* novel words are included as part of TRACE’s lexicon, i.e., assuming word learning has occurred, and infant looking times can be modelled by computing related preference for the target using Luce’s rule. Under these conditions, the simulation results provide an accurate quantitative match to the experimental data for infant looking times in block 2 of Swingley and Aslin’s (2007) study.

#### With reduced inhibition

Fig. 19 shows looking time proportions to the novel objects for neighbour and non-neighbour words when phoneme inhibition is set to 0.004, lexical inhibition to 0.02 and phoneme-to-word weights to 0.01—the parameters used previously to accommodate both White and Morgan, 2008, Mani and Plunkett, 2007 results. Under these conditions, the best fit to Swingley and Aslin’s data is obtained when no word learning has occurred and looking time is entirely driven by combined mispronunciation and novelty salience effects.

### Discussion

Simulations using TRACE offer a potential mechanism for re-interpreting the looking behaviour of the infants in Swingley and Aslin’s (2007) study. In the first block of their experiment, infants showed evidence of learning the novel non-neighbour word, but not the novel neighbour word. However, interpretation of infant behaviour in the second block of their experiment can be interpreted either as evidence for word learning, as Swingley and Aslin (2007) suggest, or as sensitivity to the severity of the mispronunciation, as in the White and Morgan (2008) study: In the case of non-neighbour trials, the novel non-neighbour (e.g., *meb*) does not activate the name-known object (e.g., *car*), given the lack of phonological overlap. On the assumption that total looking time is distributed across both images, looking time to the novel object is greater. In contrast, neighbour trials provide a mispronunciation of the name-known word (e.g., *tog-dog*), leading to partial activation of the familiar word and consequently reduced looking time to the novel object, compared to the non-neighbour trials (see Fig. 19). From this perspective, the lack of activation of the only lexical entry available (the name-known object) in the case of a non-neighbour label leads to an increased looking time to the novel object, without the need for any association between the novel label and novel object.

With default parameters, the best fit to the data is obtained when it is assumed, as do Swingley and Aslin (2007), that word learning has occurred (Fig. 19). However, in this simulation, *both* the novel neighbour and novel non-neighbour words are added as low-frequency items to TRACE’s lexicon, i.e., word learning has occurred for *both* novel words. The novel non-neighbour advantage in TRACE is driven by the interference of the familiar label in the novel neighbour condition. With reduced inhibition parameters, the best fit to the data is obtained when *no word learning* has occurred. In either case, Swingley’s and Aslin’s results are best explained in terms of a mispronunciation effect rather than an advantage for learning novel words which do not have neighbours compared to those that do.

**Fig. 20:**
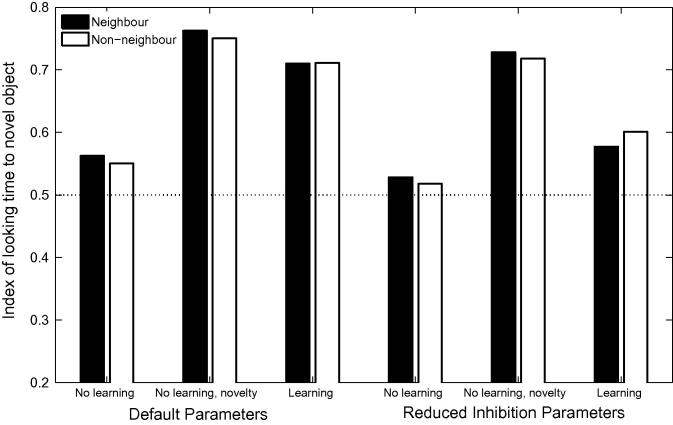
Looking preference to the novel object when the distractor (apple) has no phonological overlap to any of the novel words when TRACE is configured with default or reduced inhibition parameters. Free from any mispronunciation effects, looking times are symmetric across all simulation conditions.

If Swingley and Aslin’s findings are determined primarily by a mispronunciation effect rather than a word learning effect, then TRACE predicts that an experimental manipulation that abolishes the mispronunciation effect will result in similar performance for novel neighbour and non-neighbour words. For example, suppose that the novel objects in the infant experiments are paired with familiar objects whose labels are phonologically distinct from any of the novel labels, such as *apple*. In this experimental situation there can be no mispronunciation effect because there is no overlap between novel and familiar labels. In TRACE (see Fig. 20), responses to novel neighbour and non-neighbour words are symmetric under all simulation scenarios, with consistently strong responses in the “no learning plus novelty salience” simulations. TRACE predicts that infants will demonstrate a clearcut target preference for both novel neighbour and non-neighbour words, when novel objects are paired with familiar, but phonologically unrelated objects.

In the absence of concrete experimental evidence, it is difficult to adjudicate between Swingley and Aslin’s (2007) interpretation of their results in which “children learned the novel nonneighbors but not the novel neighbors” (ibid. p. 99) and a mispronunciation account in which both or neither novel labels are learnt successfully by their infant participants, but where the testing conditions favoured one type of label over the other. We suggest that testing infants with familiar-novel object pairs, where the familiar label is unrelated to the novel labels, will permit a resolution between these alternative explanations. For the present, Swingley and Aslin’s (2007) findings can be accommodated within a computational framework which imputes neither word learning nor lexical competition to their infant participants.

This alternative account of Swingley and Aslin’s findings can also explain the results of mutual exclusivity experiments in which novel labels are associated with novel objects (see e.g., Clark, 1987, Markman, 1989, Markman, 1990, Merriman et al., 1995). Of particular interest in this context, is the experimental finding that 16-month-old infants failed to display a mutual exclusivity effect (looking longer at the novel object) when the novel word used was a neighbour of a familiar word in the infant’s lexicon (*pok-sock*) but succeeded when the novel word was a non-neighbour (*meb*), as reported by Mather and Plunkett (2010). TRACE provides a metric of the relative novelty of a word by comparing it to the extant lexicon. This measure of lexical novelty determines the strength of the mutual exclusivity response and the likelihood of subsequently forming a new label-object association.

## General discussion

The goal of this research has been to offer a computational framework for interpreting infant word recognition and word learning. We have chosen the TRACE model of word recognition for this purpose as it has already been used to simulate a wide variety of results in the adult speech recognition literature.^14^ Can this same theoretical framework be used to account for aspects of infant speech recognition? Inspired by the work of Allopenna et al., 1998, Dahan et al., 2001a, Magnuson et al., 2007 and others, who showed that TRACE can be used to simulate the time course of adult spoken word recognition as indexed by eye movements in a visual world task, we have used TRACE to simulate experimental results from the infant word recognition literature that exploit a simplified version of the visual world task, namely the inter-modal preferential looking task (Golinkoff et al., 1987).

In a first set of simulations, a model of infant sensitivity to word mispronunciations reported by Mani and Plunkett (2007), we have shown that their results can be explained purely in terms of phonological competition between the candidate lexical items made available to the infant in the visual world task. In the TRACE simulations, word recognition is affected by both vowel and consonant mispronunciations, as indexed by “looking time” at the target item. The magnitude of the effect for both mispronunciation types is comparable, simulating Mani and Plunkett’s (2007) finding that there is a symmetry in infant sensitivity to vowel and consonant mispronunciations. However, the simulations reveal a developmental trend for onset consonant mispronunciations, which is not present for medial vowel mispronunciations. TRACE predicts an increasing sensitivity with vocabulary size to consonant mispronunciations which is not matched by vowel changes. TRACE also predicts that absence of a competing distractor in the visual world task will further enhance this asymmetry (see Fig. 5).

An asymmetry between vowels and consonants has been reported across many studies (Cutler et al., 2000, Nazzi, 2005, Nespor et al., 2003, Van Ooijen, 1996) where the prominent role of consonants for word recognition is highlighted. However, TRACE possesses a fully-specified set of phonemes where vowels and consonants are coded across the same set of features. The asymmetry observed in these simulations cannot arise from different representations or different specifications for vowels and consonants (also see Appendix A.1). Instead, the dissociation between consonant and vowel mispronunciations is driven by changes in the size and structure of TRACE lexicons, since all other parameters are kept constant across the different simulations. In the model, the asymmetry between vowel and consonant mispronunciations emerges from the increasing overrepresentation of consonants as onset phonemes relative to vowels as vocabulary size increases. Furthermore, the mispronunciations used in the simulations involve *onset consonant* changes and *medial vowel* changes. The increased sensitivity to consonant mispronunciations is explained by the increasing size of cohort competitors with vocabulary size, whereas medial vowel mispronunciations are less sensitive to changes in the number of cohort competitors.

A prediction naturally emerges from this observation: A language possessing a larger set of words with onset vowels than with onset consonants should display an increased sensitivity to onset vowel mispronunciations whereas sensitivity to medial consonant mispronunciations would remain stable with age. Although we know of no study that tests this prediction directly, a recent study by Højen and Nazzi (2009), using the name-based categorisation task to test infant sensitivity to vowel and consonant identity in newly learnt words (Nazzi, 2005), reports that Danish 20-month olds can learn minimal pairs of words that differ only on the vowel. In contrast, English and French infants can learn pairs of words that differ minimally on the consonant but fail when the word pairs differ minimally on the vowel (Havy and Nazzi, 2009, Nazzi et al., 2009). Danish is a vowel rich language compared to English and French. This enhanced sensitivity to vowel identity in Danish toddlers might emerge from a broader and more informative distribution of vowels in the developing Danish lexicon. Further empirical and typological work is needed to evaluate this claim.

The simulations yielded several novel predictions concerning the impact of lexical structure and content on infant sensitivities to phonemic boundaries and asymmetries in their sensitivity to mispronunciations of familiar words. TRACE predicts that categorical boundaries between related phoneme pairs (such as /b/–/p/) will shift when there is an imbalance in the size of lexical cohorts associated with these phoneme pairs, such that ambiguous tokens will be assimilated to the phoneme category associated with the larger cohort. For example, infant lexicons contain a disproportionately large number of /b/-initial words. TRACE predicts that ambiguous tokens of /p/ will be assimilated to /b/ just so long as this lexical imbalance remains. Since the infant lexicon is in flux, category boundaries will shift accordingly. At the lexical level, TRACE predicts that infants should be more tolerant of mispronunciations of the initial consonant of large cohort words than small cohort words. For example, the mispronunciation of [*bus*] as [^∗^*pus*] should be better tolerated than [*pig*] mispronounced as [^∗^*big*]. These predicted asymmetries are driven entirely by the structure and content of the lexicon, *not* by any asymmetries in the underlying, featural representation of the phonemes. Unfortunately, we know of no studies with infants that test these predictions. Any supportive evidence would point to an alternative processing account of phonological development driven by the accumulation of vocabulary, rather than the reorganisation of representational systems (Werker & Curtin, 2005).

TRACE also permits an evaluation of the impact of token frequency on mispronunciation sensitivity as is demonstrated in Fig. 2: increased token frequency yields enhanced mispronounced sensitivity, a finding consistent with a recent mispronunciation study with adults (White, Yee, Blumstein, & Morgan, 2013). Insofar as novel words might be considered forms with very low token frequency, TRACE has the potential to simulate sensitivity to mispronunciations of recently acquired words and/or minimal pairs. For example, a comparison of vowel and consonant mispronunciations of recently acquired words that form minimal pairs would permit a computational investigation of Nazzi’s (2005) claim that consonants are more salient for lexical acquisition than vowels.

In a second set of simulations, we used TRACE to model the impact of the severity of mispronunciations on word recognition by infants as reported by White and Morgan (2008). It was found that lexical or phonemic inhibition in TRACE must be substantially reduced in order to simulate a graded sensitivity similar to that observed by White and Morgan (2008). If lexical or phonemic competition in TRACE is left switched on (the default setting), an imbalance in the size of cohort competitors associated with the stimuli used by White and Morgan (2008) interferes with the mispronunciation effect simulated by TRACE and undermines the graded sensitivity effect.

Several independent considerations motivated switching off lexical or phonemic inhibition in these simulations. First, 19-month old infants may not yet possess a lexical network that is subject to competition effects. This claim is supported by recent experimental studies with 18- to 24-month old infants reporting the absence of cohort competition effects and semantic priming effects at 18-months with the onset of these effects delayed until 21–24 months of age (Arias-Trejo and Plunkett, 2009, Arias-Trejo and Plunkett, 2013, Mani and Plunkett, 2011). These studies point to the emergence of lexical competition effects sometime during the second half of the second year. We suggest that the 19-month old infants in White and Morgan’s (2008) study have not yet developed a lexical network with inhibitory connections. The absence of lexical competition in the model permits the observation of a graded sensitivity effect for the mispronunciations of the stimuli used in their experiment. It is noteworthy that McMurray et al. (2010) also manipulated lexical inhibition and decay parameters in TRACE to simulate aspects of language delay in SLI. It is also worth noting that Mirman et al. (2011) modulated lexical competition in their simulations of cohort competition in Wernicke’s aphasics. We do not suggest that SLI individuals, Wernicke’s aphasics and 18-month olds operate with lexicons of similar structure or content, but they may all share lowered levels of lexical inhibition.

Second, the target image in the White and Morgan (2008) study is always presented with an unfamiliar distractor, in contrast to other studies in which the distractor is a name-known object. The absence of a visually-depicted, name-known distractor object may effectively modulate the type of lexical competition occurring in infants’ mental lexicons. However, we are not persuaded that “learners [are] unwilling to accept mispronunciations as labels of known objects” (White & Morgan, 2008, p. 128). The re-analysis of Bailey and Plunkett (2002) revealed that 18-month olds exhibit a graded sensitivity to mispronunciation severity of the target label even though the distracter label is known to the infant. Finally, the removal of inhibition at the phonemic level in TRACE had similar effects to the removal of lexical inhibition. McMurray et al. (2009) suggest that phoneme-level inhibition in TRACE is incompatible with recovery from “lexical garden-paths” initiated by ambiguous phonemes early in a word.

Although 18-month-olds may lack lexical competition, the empirical evidence suggests that lexical competition effects are operative in 24-month-olds (Mani & Plunkett, 2011). This led to the prediction that 18-month olds and 24-month olds would show different response patterns to mispronunciations that are graded in severity. The re-analysis of Bailey and Plunkett (2002), in which the distractor labels possess no phonological overlap with the target, suggests that a graded sensitivity to mispronunciation severity is present in their 18-month-olds despite an slight imbalance in cohort sizes associated with the different mispronunciation conditions. In contrast, their 24-months-olds did not demonstrate such graded sensitivity. Graded sensitivity to the severity of mispronunciations with imbalanced cohort sizes can be interpreted as a signature for the absence of lexical competition. Lack of lexical competition may be manifest for words that have only recently been learnt (White et al., 2013), in certain types of aphasia (Mirman et al., 2011) or language impairment (McMurray et al., 2010), as well as for infants who have not yet developed a lexical network (Arias-Trejo and Plunkett, 2009, Arias-Trejo and Plunkett, 2013, Mani and Plunkett, 2011).

In a third set of simulations, we used TRACE to model Swingley and Aslin’s (2007) investigation of the impact of lexico-phonological competition on novel word learning. Swingley and Aslin (2007) argue that the phonological proximity of a novel word to a familiar word can inhibit word learning and that ‘lexical competition …can prevent children from using their full phonological sensitivity in judging words as novel’ [Ibid., p. 99]. We suggest an alternative account based on TRACE simulations: As White and Morgan (2008) have shown, minor mispronunciations of a familiar word do not reduce looking time at the target as much as more severe mispronunciations. Therefore, an infant will look less at a familiar object when she hears a non-neighbour of the familiar word (severe mispronunciation) than when she hears a neighbour of the familiar word (minor mispronunciation). Since looking time in a trial is almost entirely distributed between the novel and the familiar objects, White and Morgan’s results are consistent with Swingley and Aslin’s (2007) findings that infant looking time at the novel object should be longer with a non-neighbour than for a word neighbour. However, the TRACE simulations show how this pattern of behaviour may arise *in the absence of word learning and lexical competition*. Of course, increased looking time at the novel object when hearing a novel word (as opposed to a mispronunciation of a familiar word) could lead to the strengthening of an association between a novel word and a novel object, a form of mutual exclusivity based on novelty (Merriman et al., 1995), rather than cognitive (Markman, Wasow, & Hansen, 2003) or pragmatic (Diesendruck & Markson, 2001) factors. But increased looking time at the novel object need not imply that word learning has occurred or that lexical competition is operative. Hence, it could be argued that word learning through mutual exclusivity is the by-product of possessing a graded sensitivity to mispronunciation rather than the action of competitive processes that interfere with learning. This type of phonological novelty-based responding has been reported in a recent study of word learning with 16-month olds (Mather & Plunkett, 2010). TRACE offers an index of word novelty that can be used to predict looking behaviour in a preferential looking task, such as that reported by Swingley and Aslin (2007), that need not involve word learning or lexical competition.

The third set of simulations also explored the possibility that *both* novel words, neighbour and non-neighbour, were learnt by the infants in Swingley and Aslin’s study, by incorporating the novel words as low frequency items in TRACE’s lexicon. TRACE was able to mimic their findings quite accurately. The mispronunciation effect associated with the familiar object, when a novel neighbour word was presented, reduced target looking (to the novel object) compared to the novel non-neighbour condition. One way to adjudicate between Swingley and Aslin’s claim that only one word was learnt—the novel non-neighbour—and the possibility that both words were learnt, is to perform an IPL experiment in which the novel object is paired with a familiar but phonologically unrelated object, e.g., *apple*. This would eliminate any mispronunciation effect. Under these conditions, TRACE predicts a similar pattern of looking behaviour for both novel neighbour and non-neighbour words, whereas Swingley and Aslin’s account predicts that target looking will only occur for the word that has been learnt, i.e., the novel non-neighbour.

The primary goal of these simulations has been to determine whether the computational framework offered by TRACE can shed as much light on the processes of infant word recognition as it has done in the field of adult word recognition. Of course, time and space have forced us to be selective in our choice of studies to simulate. We have chosen studies where TRACE can offer an alternative theoretical interpretation of the results than that offered by the authors themselves. This does not indicate that the authors’ own interpretations are necessarily incorrect. An index of the theoretical maturity of a field, such as infant word recognition, is the degree to which computational models, i.e., precisely implemented theories, can accommodate a wide range of empirical findings. Accommodation of the model itself can then be used as an index of theoretical progress. Relatively minor modifications to the TRACE model of speech perception (McClelland & Elman, 1986) have enabled us to account for a range of findings in the infant word recognition literature. These include modification of TRACE’s lexicon to age appropriate content and manipulation of inhibitory and connection strength parameters. None of these changes constitute a departure from the general theoretical framework offered by TRACE. There are of course many other studies in the infant word recognition literature that we have not touched upon. Clearly, an adequate model of infant word recognition needs to be able to account for these findings. Here, we briefly describe another series of studies that can be readily accommodated within the framework of TRACE.

Fernald, Swingley, and Pinto (2001), expanding on a set of previous studies (Fernald et al., 1998, Swingley et al., 1999) using the IPL task, demonstrated that 18- to 24-month old infants could identify the target referent of a familiar word when only the first 300 ms of the word had been heard. Furthermore, Fernald et al. (2001) demonstrated that older infants were faster to recognise the target referent than younger infants and that this increment in speed correlated with increasing vocabulary size. These findings are readily accommodated within the TRACE framework. As demonstrated in the first set of simulations and Appendix A.2, even slight mispronunciations of word-initial consonants will activate the appropriate lexical item in TRACE. Provided the first 300 ms of the word offers unambiguous information about target identity compared to any competitors, TRACE supplemented with the Luce choice rule, will successfully identify the target referent. Increased speed of target recognition in TRACE might be achieved in a variety of ways: for example, the lexical activation parameter in TRACE could be turned up as vocabulary size increases, so as to achieve recognition threshold more speedily. However, it is also possible that increasing speed of recognition with age might itself be a by-product of increasing vocabulary size. As we have argued in the second set of simulations, increasing vocabulary size leads to greater competition motivating the need for inhibitory processes. This can be modelled in TRACE by manipulating the lexical competition parameter. Enhanced competition leads to speedier elimination of inappropriate lexical items and, therefore, faster target recognition. On this account, the acceleration in the speed of word recognition reported by Fernald et al. (2001) might be independently motivated, thereby avoiding the need to manipulate an additional parameter in the model.

## Conclusion

In the Introduction, we described two general processes that might facilitate infants in becoming efficient word recognisers. One process, which we dubbed the ‘familiarity hypothesis’, suggested that increased exposure to a given word would facilitate recognition. A second process which we called the ‘developmental hypothesis’ suggested that the need to discriminate similar sounding words would lead to more efficient recognition. We have demonstrated that TRACE can embody both of these processes: TRACE achieves more robust recognition for higher frequency words and increases in the size of the TRACE lexicon, mimicking early vocabulary development, lead to greater levels of discrimination amongst word-initial segments. In the first set of simulations, the frequency manipulations in TRACE did not impact the overall pattern of results that are imposed by manipulations of lexical size and structure (see Fig. 1). However, manipulation of phoneme-to-word connection strengths was critical for simulating word recognition skills in younger infants where it was necessary to reduce lexical competition (see Fig. 17). These outcomes warrant the assumptions that the ‘developmental hypothesis’ can be operationalised in terms of increasing levels of inhibition in TRACE as vocabulary increases in size, and that the ‘familiarity hypothesis’ can be operationalised in terms of varying the levels of connection strengths. The results of our simulations indicate not only that TRACE *can* embody these hypotheses, but that their simultaneous manipulation in TRACE seem to accurately characterise the development of infant word recognition processes. By implication, TRACE’s capacity to accommodate a range of infant word recognition results across the second year of life where the infant lexicon undergoes substantial change, points to a *continuity* in the processes and representations underlying these changes, a view that stands in contrast to theories that invoke deep reorganisation of the mechanisms that underly speech perception and word recognition in late infancy.

Finally, it should be noted that many simplifying assumptions were adopted in the simulations reported in this research. For example, word token frequencies were kept constant across age conditions. The lexicons used in the simulations were created by assessing typical vocabularies as assessed by the Oxford CDI (Hamilton et al., 2000). However, individual differences in lexicon sizes and composition would lead to a distribution of phonological sensitivities and looking patterns rather than a single uniform result in TRACE for a given age group. Moreover, the nonlinear impact of lexical competition in TRACE implies that a mean looking pattern based on a mean lexicon would not match the mean of looking patterns associated with different lexicon sizes. Fitting TRACE to individual lexicons rather than a standardised lexicon would provide yet another series of novel experimental predictions against which to evaluate the model.

## Figures and Tables

**Fig. A.21:**
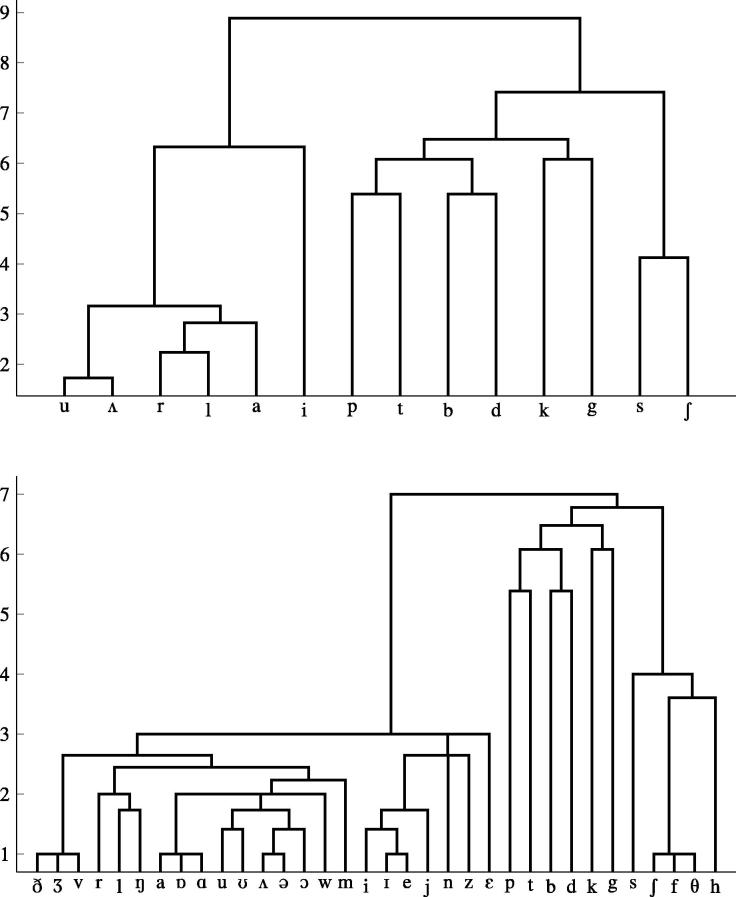
Top: initial phoneme metrics. Vowels are not strictly set apart from consonants. Bottom: present phoneme metrics. Again, vowels are not strictly set apart from consonants.

**Fig. A.22:**
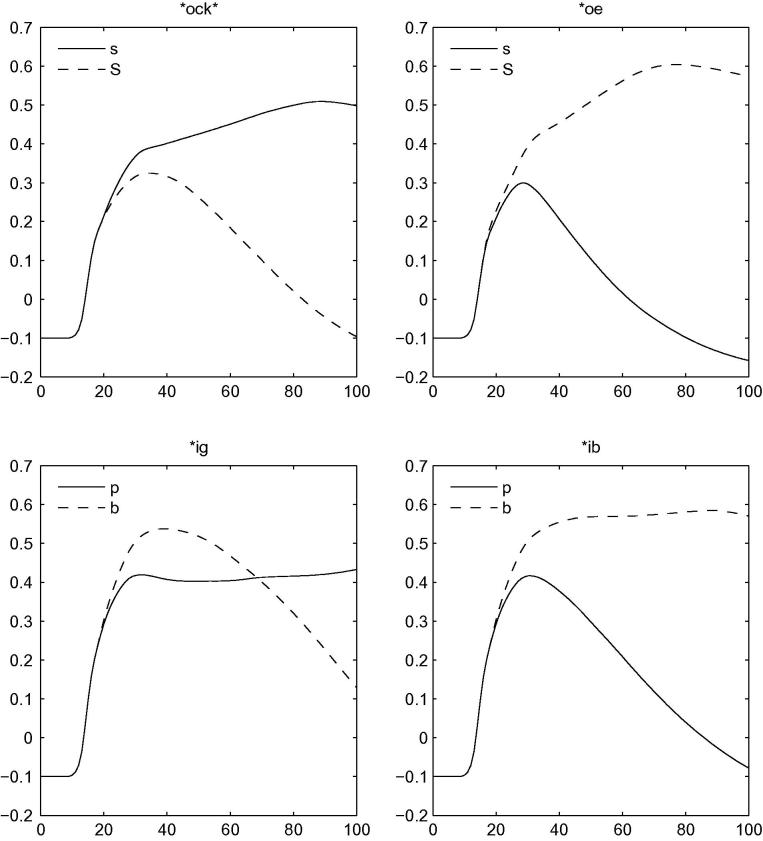
Ganong effect with initial ambiguity.

**Fig. A.23:**
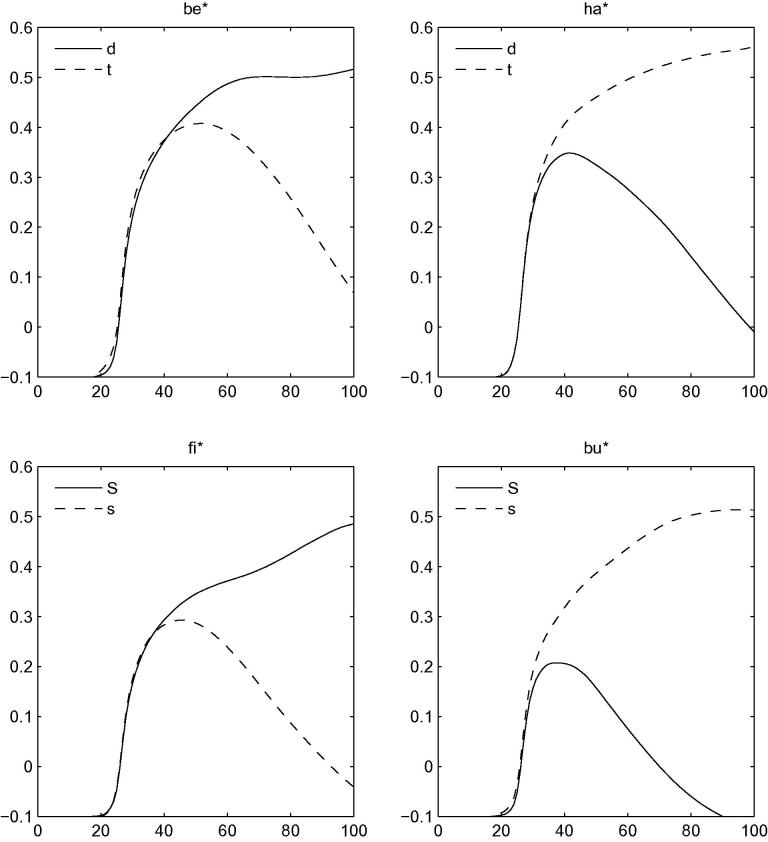
Ganong effect with final ambiguity.

**Fig. A.24:**
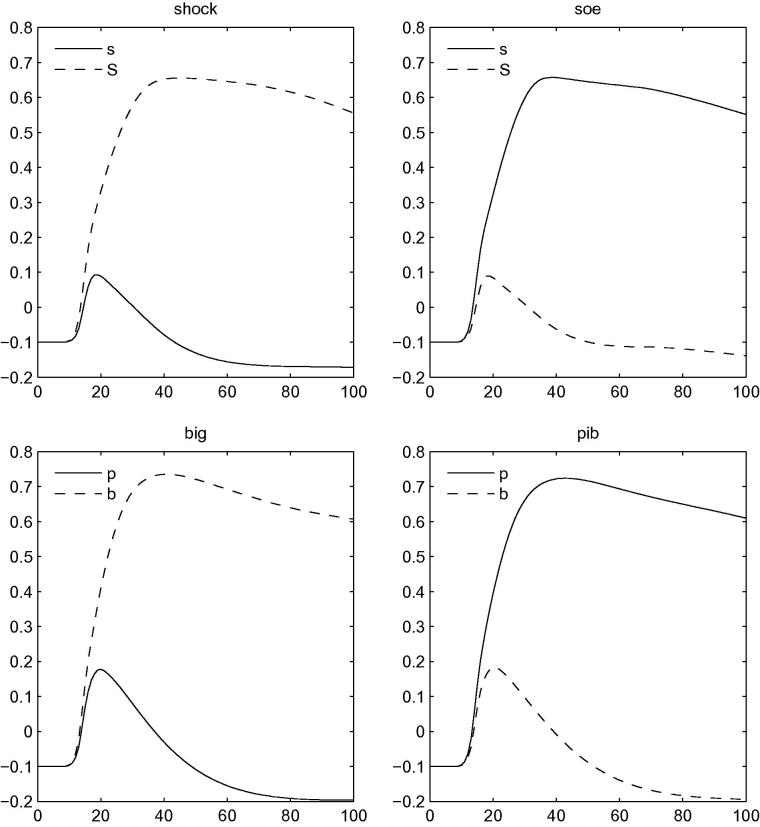
Ganong effect with a different initial phoneme (no ambiguity).

**Fig. A.25:**
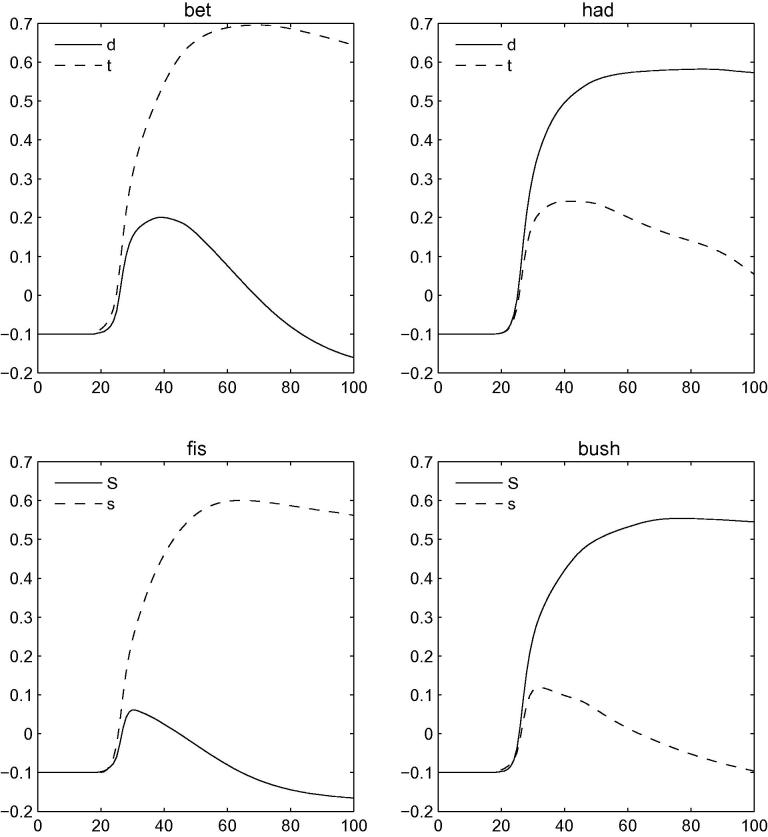
Ganong effect with a different final phoneme (no ambiguity).

**Table B.8:** Lexicon at 15 months. Frequencies are raw counts on the Manchester corpora (Theakston et al., 2001) from the CHILDES database.

Word	Frequency	Word	Frequency	Word	Frequency	Word	Frequency
baby	3893	brush	226	hair	1254	sheep	557
ball	638	bus	608	hat	1171	shoe	282
balloon	248	car	5051	highchair	1	sock	190
banana	382	cat	1079	horse	1495	spoon	304
bath	594	coat	172	key	240	stairs	125
bathtub	1	cow	1365	milk	935	teddybear	1
bear	475	cup	610	moo	17	telephone	116
bed	1021	daddy	301	mouth	662	toothbrush	25
bib	104	dog	1011	mummy	1000	tree	471
bike	234	door	1130	nappy	1	woof	2
bird	223	drink	1203	nose	876		
book	1365	duck	465	pig	726		
boots	113	flower	146	quack	1		

**Table B.9:** Lexicon at 18 months. Frequencies are raw counts on the Manchester corpora (Theakston et al., 2001) from the CHILDES database.

Word	Frequency	Word	Frequency	Word	Frequency	Word	Frequency
aeroplane	229	byebye	1	hair	1254	sheep	557
apple	690	cake	1104	hand	898	shoe	282
arm	337	car	5051	happy	1	slide	357
baby	3893	cat	1079	hat	1171	sock	190
ball	638	chair	691	head	1200	spoon	304
balloon	248	cheese	540	hoover	45	stairs	125
banana	382	chicken	664	horse	1495	Sun	201
bath	594	tchoo tchoo	1	house	1939	swing	94
bathroom	1	clock	131	juice	702	table	628
bathtub	1	coat	172	key	240	teddybear	1
bear	475	cockadoodledo	1	kitchen	245	telephone	116
bed	1021	cot	149	leg	473	television	1
bee	101	cow	1365	light	216	tiger	1100
bellybutton	1	cup	610	lion	515	toast	252
bib	104	daddy	301	lorry	436	toe	200
bicycle	44	dish	32	meow	1	tongue	49
bike	234	dog	1011	milk	935	tooth	7
bin	241	doll	362	monkey	893	toothbrush	25
bird	223	door	1130	moo	17	towel	125
biscuit	388	drink	1203	moon	87	toy	225
block	47	duck	465	mouse	250	train	3442
boat	793	ear	209	mouth	662	tree	471
book	1365	elephant	974	nose	876	trousers	576
boots	113	eye	310	ouch	197	truck	905
bottle	358	finger	328	outside	538	tummy	436
bowl	118	fish	946	pen	254	vacuum	4
bread	373	flower	146	pig	726	vroom	4
brick	457	foot	545	plate	254	watch	887
brush	226	fridge	123	pushchair	1	water	1148
bubble	15	frog	189	pyjamas	1	window	392
bunny	65	garden	306	quack	1	woof	2
bus	608	grandma	46	rabbit	515	yum	203
butterfly	130	grandpa	16	refrigerator	1		

**Table B.10:** Lexicon at 24 months. Frequencies are raw counts on the Manchester corpora (Theakston et al., 2001) from the CHILDES database, part 1.

Word	Frequency	Word	Frequency	Word	Frequency	Word	Frequency
aeroplane	229	coffee	164	leg	473	shirt	138
animal	253	comb	70	light	216	shoe	282
apple	690	cooker	58	lion	515	shop	565
arm	337	cot	149	living room	1	shorts	59
baby	3893	cow	1365	lorry	436	sink	55
ball	638	cup	610	man	2074	sky	237
balloon	248	daddy	301	meat	180	slide	357
banana	382	dish	32	medicine	167	snow	139
bath	594	dog	1011	meow	1	soap	69
bathroom	1	doll	362	milk	935	sock	190
bathtub	1	donkey	88	money	596	sofa	63
beach	134	door	1130	monkey	893	spaghetti	93
bear	475	drawer	17	moo	17	specs	1
bed	1021	dress	726	moon	87	spider	400
bee	101	drink	1203	motorbike	77	spoon	304
bellybutton	1	duck	465	mouse	250	squirrel	21
bib	104	ear	209	mouth	662	stairs	125
bicycle	44	egg	1052	nail	78	star	198
bike	234	elephant	974	nappy	1	stone	50
bin	241	eye	310	necklace	37	stove	2
bird	223	face	638	nose	876	sun	201
biscuit	388	finger	328	orange	1387	sweater	16
block	47	fireengine	1	ouch	197	sweets	54
boat	793	fish	946	outside	538	swing	94
book	1365	flower	146	oven	99	table	628
boots	113	food	727	owl	40	tea	1377
bottle	358	foot	545	paper	460	teddybear	1
bowl	118	fork	117	park	324	telephone	116
box	1946	fridge	123	party	284	television	1
boy	1394	frog	189	pasta	46	tiger	1100
bread	373	garage	432	peas	180	toast	252

**Table B.11:** Lexicon at 24 months, part 2.

Word	Frequency	Word	Frequency	Word	Frequency	Word	Frequency
brick	457	garden	306	pen	254	toe	200
brush	226	giraffe	485	penguin	499	tongue	49
bubble	15	girl	1333	people	583	tooth	7
bucket	165	glass	81	picture	600	toothbrush	25
bunny	65	glasses	115	pig	726	towel	125
bus	608	grandma	46	pillow	62	toy	225
butter	70	grandpa	16	pizza	143	train	3442
butterfly	130	hair	1254	plant	30	tree	471
button	142	hammer	234	plate	254	trousers	576
byebye	1	hand	898	pool	192	truck	905
cake	1104	hat	1171	potty	1	tummy	436
car	5051	head	1200	puppy	66	vacuum	4
carrot	107	highchair	1	purse	30	vroom	4
cat	1079	hoover	45	pushchair	1	wall	299
cereal	4	horse	1495	pyjamas	1	watch	887
chair	691	house	1939	quack	1	water	1148
cheek	19	jacket	31	rabbit	515	window	392
cheese	540	juice	702	radio	39	woof	2
chicken	664	jumper	135	rain	176	work	905
chips	676	key	240	refrigerator	1	yum	203
choo choo	1	kitchen	245	rubbish	101	zip	45
clock	131	knee	496	scissors	53		
coat	172	lady	756	settee	142		
cockadoodledo	1	lamb	137	sheep	557		

## Appendix A TRACE with the expanded set of phonemes

In order to simulate infant word learning experiments, we expanded the set of phonemes so that all words in the typical infant lexicon can be part of the TRACE lexicon, as well as all words presented to the model. The original TRACE model (McClelland & Elman, 1986) used 14 phonemes. After adding supplementary phonemes, the total count reaches 34. This expansion of the phoneme set results in a denser phoneme space than the original TRACE model. We first present clustering analyses of both the original and the new phoneme sets. In order to evaluate the impact of having a more crowded phonemic space on the behaviour of the model, we have replicated a series of classic TRACE simulations with the new phoneme set.

### A.1 Phoneme metrics

The original TRACE model offered a series of experimental predictions in the field of speech perception, and accurately described a large set of experimental findings. It is pertinent to ask whether the modified set of phonemes used in the current simulations to model infant word recognition impacts dynamical behaviour in such a way as to undermine TRACE’s capacity to simulate human performance. To facilitate comparison, we conducted the same clustering analyses on both the original phoneme set and on the expanded phoneme set.

The feature vectors representing individual phonemes in the original and expanded phoneme sets were analysed using an unsupervised clustering algorithm. We applied a standard single-linkage clustering method, with Euclidian distance as the similarity metric. This algorithm creates a number of clusters in which the stimuli in each cluster are close together in Euclidian space. The total number of clusters obtained depends on the definition of a distance threshold such that a small threshold leads to many clusters and a large threshold leads to a small number of clusters. Dendrograms are a visually transparent representation of the number of clusters produced by this algorithm (Sneath, 1957). The number of clusters formed corresponds to the number of vertical lines for a specified threshold.

Fig. A.21 depicts the clustering dendrograms corresponding to the original TRACE set and the expanded phoneme set, respectively. Vowels are labelled “v” and consonants “c”. A natural split into two clusters fails to offer a clear partition between vowels and consonants, in both phoneme sets. Although in each case, one branch of the dendritic tree is populated solely by consonants, the other branch contains a mixture of vowels and consonants. In neither of the phoneme inventories is there an inherent partitioning of the feature space that facilitates a clearcut distinction between vowels and consonants.

We also measure the density of packing of vowels and consonants in each feature space by measuring the mean Euclidean distance between vowels, and between consonants, respectively. For the original phoneme inventory, vowels are more densely packed (mean distance: 5.37) than consonants (mean distance: 9.26). Likewise, for the expanded phoneme inventory vowels are more densely packed (mean distance; 4.29) than consonants (mean distance: 8.30). From a structural perspective, we can conclude that the new phoneme set respects the structure of the original phoneme set, even though it is more tightly packed.

### A.2 Replication of standard simulations

Most of the simulations reported in this paper evaluate TRACE’s sensitivity to mispronunciations of familiar words. Therefore, we report how TRACE responds to mispronunciations when tested with the expanded phonemic inventory and infant lexicon (24 months), given its greater degree of packing in the phonemic feature space. These simulations correspond to some of the original simulations carried out by McClelland and Elman (1986, p. 24–28) to test TRACE’s capacity to simulate the Ganong effect (Ganong, 1980). In particular, we examine TRACE’s capacity to simulate the phoneme restoration effect with ambiguous phonemes in both word-initial and word-final position, using the new lexicon. We also examine whether phoneme restoration effects *fail* to occur with the new lexicon when unambiguous but incorrect phonemes are used in word-initial and word-final position, as reported by Ganong (1980) and simulated in the original TRACE model.

#### A.2.1 Ambiguous segments in word-initial position

Fig. A.22 plots the time course of phoneme activations in TRACE with the infant lexicon when ambiguous phonemes are used in word-initial position. The top left and right panels shows phoneme activations for /s/ and /ʃ/ when an ambiguous segment midway between /s/ and /ʃ/ is embedded in the sequences ${}^{*}\mathrm{ock}$ or ${}^{*}\mathrm{oe}$.^15^ Once the target word has been activated in TRACE, phoneme restoration occurs and /ʃ/ is appropriately disambiguated from /s/, in accordance with the most likely lexical context.

Similarly, the bottom panels of Fig. A.22, show phoneme restoration effects in TRACE when the word-initial ambiguous phoneme is midway between /p/ and /b/. In the bottom-left panel, the sequence [${}^{*}\mathrm{ig}$]^16^ lead to a higher activation of /p/ whereas /b/ becomes more active for the sequence [${}^{*}\mathrm{ib}$].^17^ These findings highlight the role of lexical influences on phoneme perception thought to underlie the Ganong effect and confirms that the expanded set of phonemes and infant lexicon yield similar phoneme restoration effects as the original TRACE simulations.

#### A.2.2 Ambiguous segments in word-final position

Ambiguous phonemes can also occur at the end of words. Fig. A.23 plots the time course of phoneme activations in TRACE with the infant lexicon when ambiguous phonemes are used in word-final position. The top left and right panels shows phoneme activations for /d/ and /t/ when an ambiguous segment midway between them is embedded in the sequences [${\mathrm{be}}^{*}$] or [${\mathrm{ha}}^{*}$].^18^ Once the target word has been activated in TRACE, phoneme restoration occurs and /d/ is appropriately disambiguated from /t/, in accordance with the most likely lexical context. In the bottom panels of Fig. A.23, the ambiguous word-final segment, midway between /s/ and /ʃ/, is disambiguated once the target word is fully activated. When the target word is *fish*, the activation of the phoneme /ʃ/ wins over /s/ whereas the opposite pattern is observed when the target word is *bus*. These findings demonstrate that phoneme restoration effects are robust in word-final position, emulating the behaviour of the original model.

#### A.2.3 Unambiguous mispronunciations in word-initial position

Ganong (1980) reports that phoneme restoration effects do not occur for unambiguous mispronunciations of familiar words, neither in word-initial nor word-final position. The original TRACE model simulates this limitation on top-down, lexically-driven effects. Fig. A.24 displays the time course of phoneme activations in TRACE with the infant lexicon when unambiguous but incorrect phonemes are used in word-initial position. The top left and right panels shows phoneme activations for /ʃ/ and /s/ when embedded in the sequences [*shock*] or [*sue*]. The phoneme /ʃ/ is activated to a much higher level than /s/, despite the fact that *sock* is a word in the TRACE lexicon. Symmetrically, in the top right panel, when *sue* is used as an input, /s/ is activated more than /ʃ/, even though *shoe* is an existing lexical entry. This prevalence of bottom-up effects over lexical effects is valid for other phoneme pairs. As a further illustration, the bottom panels of Fig. A.24 show that when *big* is presented to the network, /b/ is activated more than /p/ even though *pig* is a lexical entry (and *big* is not). Similarly, *pib* activates /p/ more than /b/ despite the fact that *bib* is part of the lexicon.

#### A.2.4 Unambiguous mispronunciations in word-final position

Finally, Fig. A.25 displays the time course of phoneme activations in TRACE with the infant lexicon when unambiguous but incorrect phonemes are used in word-final position. The top left and right panels shows phoneme activations for /t/ and /d/ when embedded in the sequences [*bet*] or [*had*]. The phoneme /t/ is activated to a much higher level than /d/, despite the fact that *bed* is a word in the TRACE lexicon. Likewise, when *had* is used as an input, /d/ is activated more than /t/, even though *hat* is an existing lexical entry. The lower panels show corresponding patterns of activation for the phonemes /s/ and /ʃ/ in the sequences [*fis*] and [*bush*]. Top-down, lexical effects are unable to overcome the bottom-up activation of the unambiguous but contextually inappropriate phonemes.

In summary, ambiguous phonemes are disambiguated via lexical effects feeding-back onto the phoneme level. In contrast, non-ambiguous phonemes activate their corresponding phoneme node at the phoneme level, overruling potential lexical effects. Both sets of results for word-initial and word-final manipulations correspond accurately to the original TRACE simulations and confirm the validity of TRACE when geared with an expanded phoneme set and infant lexicon.

## Appendix B Lexicons used in the simulations

See Table B.8, Table B.9, Table B.10, Table B.11.

## Footnotes

1 We justify this claim with additional analyses presented in Appendix A.1.

2 This threshold was used so that, typically, infants know most of the words used in the experiment. However, creating a mean lexicon is not equivalent to averaging simulations over many infants, each possessing a different lexicon size and structure. This is both a limitation of the current model and an avenue for further research, aimed at modelling individual differences.

3 Thanks to Ōiwi Parker-Jones for help in assigning feature values for phonemes not present in the original TRACE model.

4 2500 ms minus 367 ms needed to elicit language mediated eye-movements (Swingley & Aslin, 2000).

5 In the original experiments, a naming effect occurs when a significant difference between pre-naming and post-naming target looking is observed. In the simulations, since only post-naming phases are modelled, this translates into whether looking times exceed 50%.

6 This duration is rounded up from 19.38 ms for purposes of exposition. Consequently, the peak looking time reported for the model in the correct pronunciation condition is slightly longer than for infants.

7 Removal of lexical-phonemic feedback is achieved simply by setting these connections to zero.

8 Note that neighbourhood densities across mispronunciation conditions are not significantly different.

9 Analysis of a dense recording at 30 months of age, the Haggerty corpus (Haggerty, 1929), revealed that /b/-onset words (89 words) are almost twice as numerous as /p/-onset words (48 words).

10 For comparison, the value commonly used to model adult sensitivities to mispronunciations is *C* = 0.03 (see for example Allopenna et al., 1998) which means inhibition in the word layer is 300 times stronger than the value used here.

11 Though Höhle, van de Vijver, and Weissenborn (2006) reported that 19-month olds with later low language performance showed longer looking times in mispronunciation conditions than in correct pronunciation trials, in contrast with infants with normal later language performance. Note, however, that the distractors used in their study matched the onset of the mispronounced words in some cases whereas in other cases they did not.

12 Normal controls show graded sensitivity to a non-word prime determined by the featural distance of the non-word from the correct prime. For example, *gat* produces stronger priming of *dog* than does *wat*, presumably because it is closer in phonological distance to *cat*.

13 We thank an anonymous reviewer for this suggestion.

14 We do not mean to imply that other computational models cannot be used for this purpose. Hopefully, this work will promote the evaluation of competing computational frameworks against the infant speech recognition literature.
